# Multimodal Deep Learning with Routine Clinical Data for Recurrence Risk Stratification in HR^+^/HER2^−^ Early Breast Cancer

**DOI:** 10.34133/research.1136

**Published:** 2026-03-30

**Authors:** Xiaoyan Wu, Hong Liu, Jingyan Liu, Bing’an Mu, Jianfei Li, Siyu Wang, Fengling Li, Xunxi Lu, Jie Chen, Yulan Peng, Yuhao Yi, Jiancheng Lv, Hong Bu

**Affiliations:** ^1^ Department of Pathology, West China Hospital, Sichuan University, Chengdu, China.; ^2^ Institute of Clinical Pathology, West China Hospital, Sichuan University, Chengdu, China.; ^3^ College of Computer Science, Sichuan University, Chengdu, China.; ^4^ Department of Medical Ultrasound, West China Hospital, Sichuan University, Chengdu, China.; ^5^ College of Computer Science and Technology, Chongqing University of Posts and Telecommunications, Chongqing, China.

## Abstract

Hormone receptor-positive/human epidermal growth factor receptor 2-negative (HR^+^/HER2^−^) early breast cancer (EBC) patients face long-term recurrence risk despite standard treatment. Current prognostic tools relying on clinicopathological factors or multigene assays have limited accuracy or accessibility. In this study, we developed a multimodal recurrence risk prediction (MRRP) model integrating routinely available clinical data, including whole-slide images (WISs), ultrasound (US) imaging and diagnostic reports, and structured clinical parameters. The MRRP model employs a hierarchical transformer-based fusion framework with innovative intra- and intermodality cross-attention mechanisms to dynamically integrate diverse feature representations. Using a well-curated cohort of 768 HR^+^/HER2^−^ EBC patients with long-term follow-up, MRRP demonstrated superior prognostic performance (C-index = 0.840) compared to single-modality models, with robust time-dependent AUCs exceeding 0.85 at 3, 5, and 7 years. Ablation studies highlighted the central role of pathology features and the complementary value of US and clinical data. We further validated the optimal query selection strategies and evaluated different pretrained encoders, revealing complex modality interactions. To address real-world challenges of missing modality data, a learnable compensation mechanism was implemented, improving model robustness. Our study provides a clinically practical, AI-driven tool for precise risk stratification in HR^+^/HER2^−^ EBC patients, facilitating individualized treatment and surveillance decisions without reliance on costly multi-omics data.

## Introduction

Hormone receptor-positive/human epidermal growth factor receptor 2-negative (HR^+^/HER2^−^) breast cancer is the most common subtype of breast cancer, accounting for over 70% of cases [1]. Although early-stage patients generally achieve favorable prognoses through surgery combined with adjuvant therapy, approximately 20% to 30% of patients still face long-term risk of recurrence [2]. Therefore, precise assessment of recurrence risk in HR^+^/HER2^−^ breast cancer patients remains a critical challenge in the era of precision medicine. Currently, clinical prediction tools mainly rely on clinicopathological factors and multigene assays (such as Oncotype DX [3–5], MammaPrint [6,7]), but their predictive performance remains suboptimal, with most models exhibiting concordance indices (C-index) below 0.7 [3,8,9]. Moreover, multigene assays are costly and have limited accessibility, which restricts their widespread clinical application. In parallel, several artificial intelligence (AI)-based models using routine hematoxylin and eosin (H&E)-stained whole-slide images (WSIs) have shown that digital histopathology alone can provide independent prognostic information in breast cancer. For example, Wang et al. [10] developed the DeepGrade model to improve risk stratification among Nottingham histological grade 2 tumors using deep learning on WSIs, and the CE-IVD-marked (Conformité Européenne-In Vitro Diagnostic-marked) Stratipath Breast solution has been validated for WSI-based risk stratification in large, independent breast cancer cohorts and has shown considerable agreement with an established multigene assay in estrogen receptor–positive (ER^+^)/HER2^−^ early breast cancer (EBC) [11,12]. Thus, developing a practical and effective AI-based risk prediction model that leverages routinely collected clinical data, including pathology images, imaging examinations, and structured clinical information, is of great importance for optimizing treatment decisions and follow-up strategies.

In recent years, AI has demonstrated tremendous potential in healthcare, particularly in integrating heterogeneous multi-source medical data to build multimodal predictive models with substantial progress [13–16]. Such models leverage the complementary nature of different data modalities to achieve more accurate clinical prognostic predictions compared to single-modality approaches [17–20]. For example, in breast cancer prognosis prediction, the CIMPTGV model developed by Zhang’s team [21] integrates clinical information, multidimensional immunohistochemistry, metabolomics, pathology, transcriptomics, genomics, and copy number variations, achieving excellent performance with a C-index of 0.869. However, most existing multimodal studies rely heavily on expensive and time-consuming omics data, while research on multimodal risk prediction models based solely on routinely available clinical data (such as digital pathology images, US imaging, and diagnostic text reports) remains limited. Developing such clinically practical models faces multiple challenges: First, heterogeneous data from different departments and devices suffer from inconsistent standards and decentralized storage, complicating efficient cross-department data integration; second, the heterogeneity of each modality requires tailored feature encoders, whose performance directly affects prediction accuracy; third, designing effective multimodal fusion strategies is complex, with cross-modal interaction mechanisms posing core technical challenges; lastly, although missing data modalities are common in real-world clinical practice, most existing studies train and validate multimodal models only on patients with complete modality data, which effectively reduces the usable sample size and may compromise model performance and generalizability [22]. These factors collectively hinder the clinical implementation and widespread application of multimodal risk prediction models.

To meet clinical needs and overcome the challenges described above, we developed a multimodal recurrence risk prediction (MRRP) model for patients with HR^+^/HER2^−^ EBC. The model was built using low-cost and easily accessible clinical data from multiple sources, including pathomics, US radiomics, and clinical information. In total, 768 patients were included. The dataset contained WSI, grayscale US images, US diagnostic reports, and structured clinical information. We proposed an innovative hierarchical fusion framework to address the difficulty of integrating these different types of data. In the pathology branch, a cross-attention mechanism was used to combine deep learning features from a pretrained encoder with morphological and spatial topological features derived from the sc-MTOP (single-cell morphological and topological profiling) framework [23], which uses a Hover-Net model pretrained on the public PanNuke dataset to generate single-cell-level nuclear segmentation and cell-type labels for subsequent morphological and spatial topology profiling. In the US branch, we integrated deep image features obtained from a self-trained encoder, radiomics features extracted using the PyRadiomics library [24], and text embeddings generated by a large language model to capture the semantic content of diagnostic reports. Pathomics and US radiomics features were then merged, and the combined features were further integrated with structured clinical information processed by a learnable encoder through cross-attention. The MRRP model was able to capture complementary information from different modalities and showed excellent performance in predicting recurrence risk. This approach offers an economical, efficient, and clinically feasible tool for risk stratification in HR^+^/HER2^−^ EBC.

## Results

### Characteristics of the HR^+^/HER2^−^ EBC multimodal dataset and construction of the MRRP framework

This study included a total of 768 eligible patients with HR^+^/HER2^−^ EBC treated at West China Hospital (WCH), Sichuan University, hereafter referred to as the WCH cohort. The patient selection process is summarized in Fig. S1, and detailed inclusion and exclusion criteria are provided in the data collection section of Materials and Methods. The median follow-up time was 88 months (range, 17 to 174 months), and the median age was 47 years (range, 25 to 78 years). Histological grade showed that grade 2 accounted for the largest proportion (57.8%, 444/768), and 57.9% (445/768) of patients had lymph node metastasis. During the entire follow-up period, 125 recurrence or metastasis events were observed (16.2%). Kaplan–Meier analysis revealed a 10-year recurrence-free survival (RFS) rate of 81.3% [95% confidence interval (CI), 78.1% to 84.5%]. The distribution of recurrent and nonrecurrent patients across subgroups and detailed clinical characteristics are presented in Table S1.

Based on the WCH breast cancer specialty cohort, we established a multimodal dataset for predicting recurrence risk in HR^+^/HER2^−^ EBC. This dataset integrated 4 commonly collected data types in clinical practice: high-resolution digitized whole-slide pathology images, standardized preoperative US images, detailed US diagnostic report texts, and structured clinicopathological parameters (including patient age, tumor size, histological grade, lymph node status, and hormone receptor expression levels). To ensure data quality, all enrolled cases underwent a strict standardized collection process and professional quality control, as detailed in Fig. S1. The dataset was randomly divided into training and test sets at a ratio of 5:1 with stratification to maintain balanced sample distribution.

**Fig. 1.**
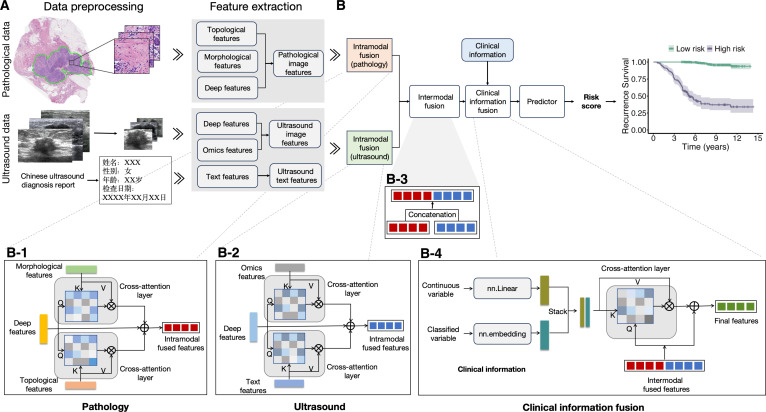
Workflow of the MRRP model for predicting recurrence risk in HR^+^/HER2^−^ EBC. (A) Preprocessing of pathological WSI and US data to extract modality-specific features. (B) Multimodal information fusion and patient risk stratification analysis. (B-1) Intramodality fusion in pathology: dynamic integration of pathological image features (deep features, morphological features, topological features) using a cross-attention mechanism. (B-2) Intramodality fusion in US: dynamic integration of US features (deep features, radiomic features, text features) using a cross-attention mechanism. (B-3) Intermodality feature fusion: concatenation of fused pathological image features and US features to form intermodality fused features. (B-4) Patient clinical information fusion module: deep interaction between intermodality fused features and structured clinical information via cross-attention, followed by residual connection of the cross-attention output with the original intermodality fused features.

**Fig. 2.**
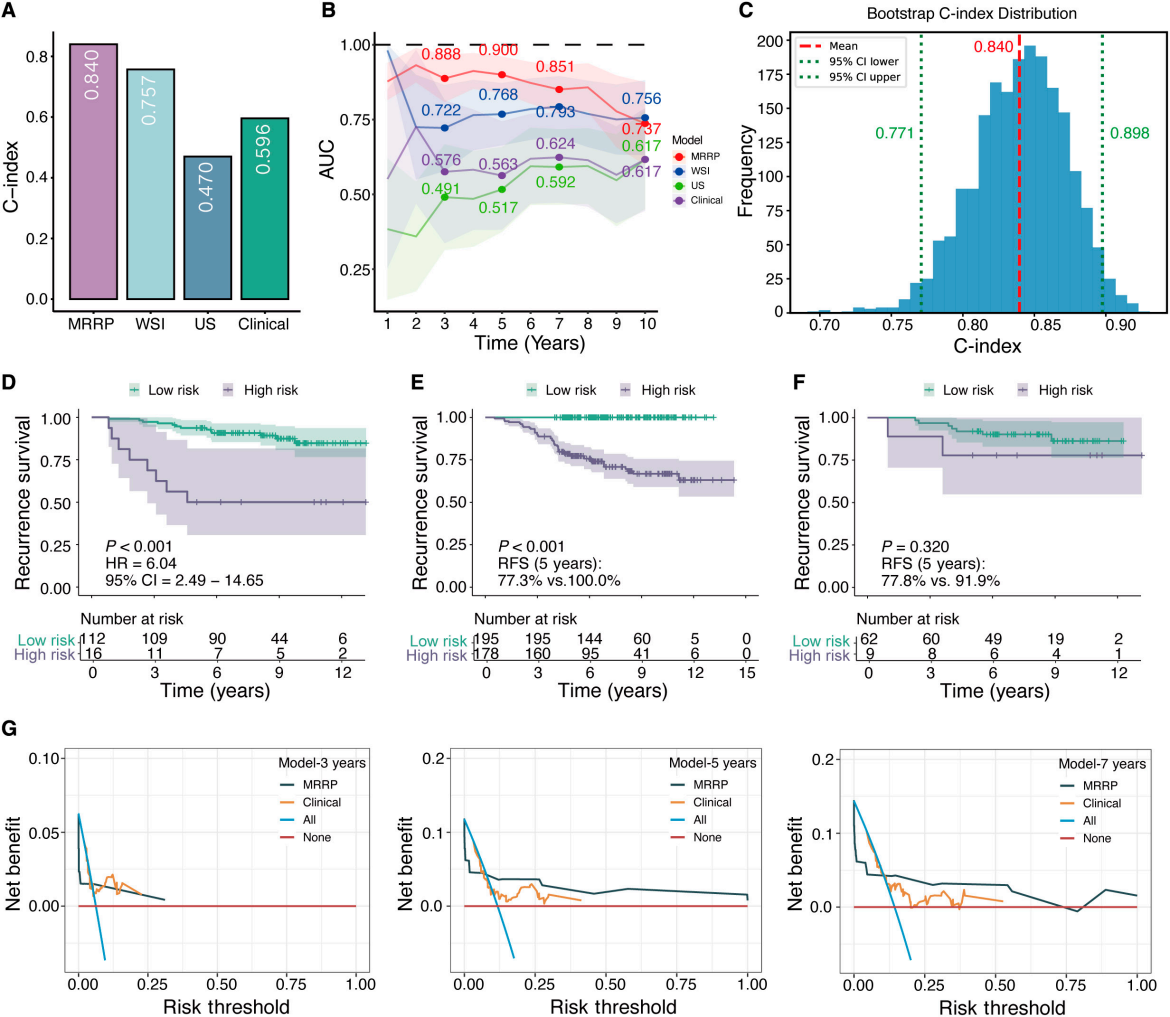
Performance of the MRRP model. (A and B) Comparison of the MRRP model with single-modality pathology, US, and clinical parameter models in predicting recurrence risk. (C) Stability assessment of model performance using 2,000 bootstrap resampling iterations. (D) Kaplan–Meier curves depicting RFS based on MRRP predictions. Patients were stratified into high-risk (purple) and low-risk (green) groups using the median MRRP score as the cutoff. Differences between groups were assessed by the 2-sided log-rank test (*P* < 0.05). (E) Kaplan–Meier curves for RFS based on MRRP stratified risk groups in grade 2 patients in the training set. (F) Kaplan–Meier curves for RFS based on MRRP stratified risk groups in grade 2 patients in the test set. (G) Decision–curve analysis (DCA) curves at 3, 5, and 7 years evaluating the net benefit of the MRRP score across a range of threshold probabilities, compared with the clinical model and the treat-all and treat-none strategies.

**Fig. 3.**
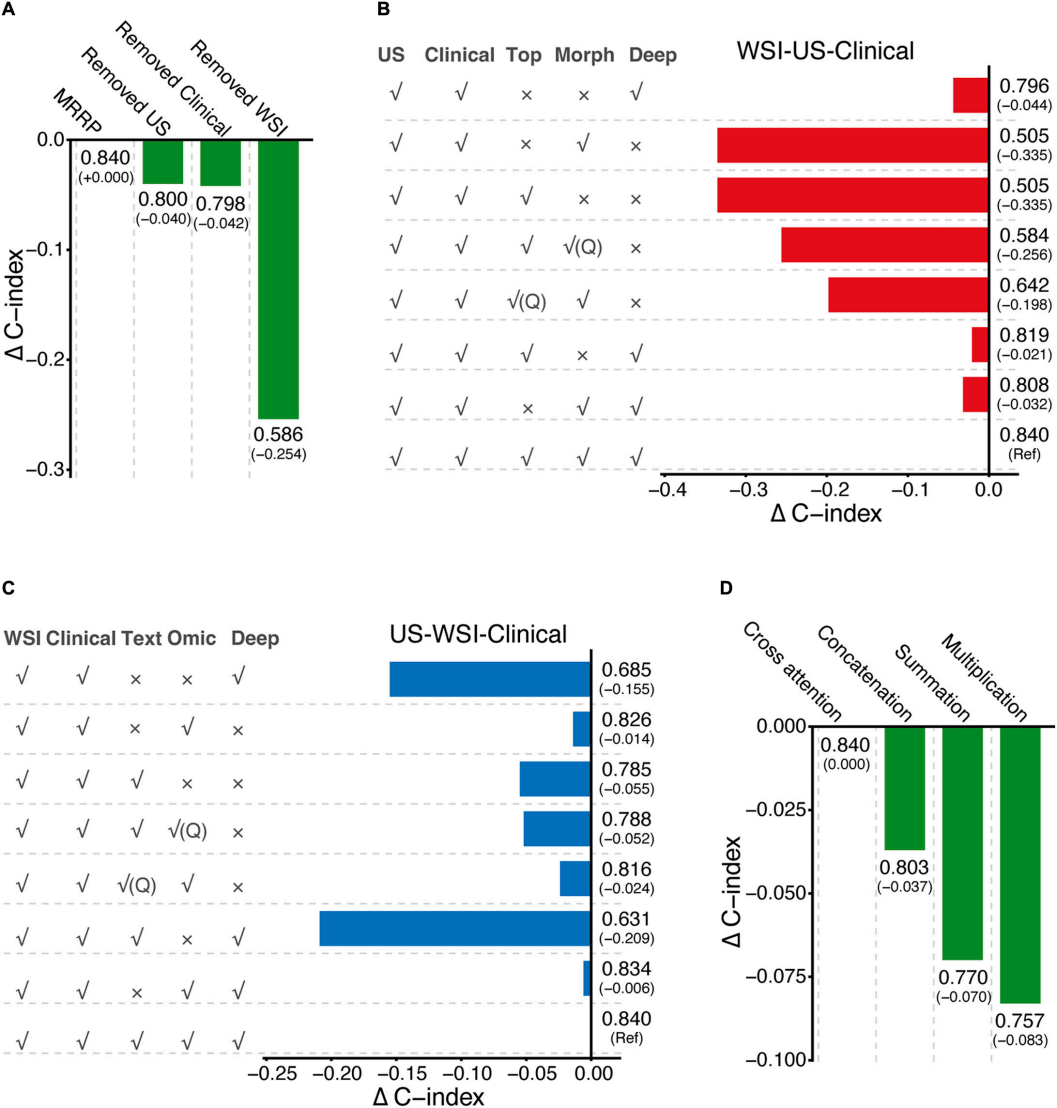
Impact of different modalities and intramodality features on MRRP model performance. (A) Bar chart showing the C-index and its changes on the test set for the full MRRP model and models with one modality removed. (B) Bar chart showing the C-index and its changes on the test set for the MRRP model and models with different pathology modality features removed sequentially. (C) Bar chart showing the C-index and its changes on the test set for the MRRP model and models with different US modality features removed sequentially. (D) Bar chart showing the C-index and its changes on the test set for MRRP models using different intermodality fusion strategies.

**Fig. 4.**
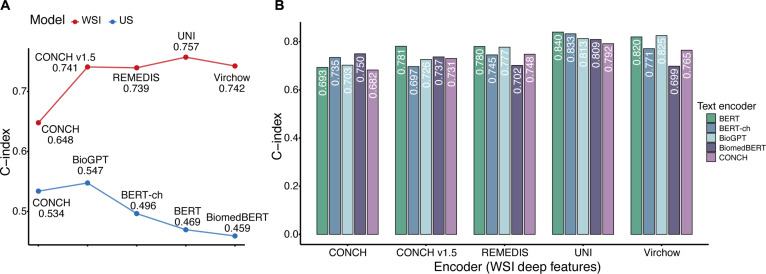
Impact of pretrained encoders on MRRP model performance. (A) Model performance of different pretrained encoders in pathology single-modality (red line) and US single-modality (blue line). (B) Performance of different combinations of pretrained encoders within the MRRP model.

During model development, a staged training strategy was adopted. First, pretrained feature encoders were used to extract modality-specific representations from pathology and US data. For pathology images, we obtained deep features, morphological features, and topological features. For US data, image deep features, quantitative radiomics features, and text semantic features were extracted (Fig. 1A). Based on these, we developed the MRRP prediction model with hierarchical fusion capabilities. The core of the framework is hierarchical fusion: intramodality fusion dynamically integrates pathology image features (deep, morphological, topological) (Fig. 1B-1) and US features (deep, radiomics, text) (Fig. 1B-2) using cross-attention mechanisms; then intermodality fusion concatenates the fused pathology and US features into cross-modality fusion features (Fig. 1B-3). These features are further deeply integrated with structured clinical information processed by a learnable encoder via a cross-attention mechanism. The output features are combined with the original intermodality fusion features through a residual connection (Fig. 1B-4), resulting in a comprehensive predictive representation for risk prediction and stratification analysis. The detailed model framework is shown in Fig. 1.

### Performance of the MRRP model

To overcome the limitations of traditional single-modality prediction models, we innovatively developed the MRRP, a multimodal recurrence risk prediction model integrating pathomics, US radiomics, and clinical parameters. In the test set, the MRRP model yielded a C-index of 0.840, representing improvements of 11%, 79%, and 41% over the pathology-only model (0.757), US-only model (0.470), and clinical parameter model (0.596), respectively (Fig. 2A). Moreover, the time-dependent area under the curve (AUC) for the MRRP model at 3, 5, and 7 years was 0.888, 0.900, and 0.851, respectively (Fig. 2B). To verify the robustness of these results, we performed 2,000 bootstrap resampling iterations to evaluate model performance on the test set. The coefficient of variation for the C-index estimate was 4.43%, and the 95% CI included the original test set result (original C-index 0.840 ∈ 95% CI [0.771, 0.898]) (Fig. 2C), confirming the stability of the model’s performance. In addition, we conducted 10 repetitions of 5-fold cross-validation with different random seeds, yielding an overall mean C-index of 0.816, with the per-repetition mean C-index ranging from 0.803 to 0.839 across held-out folds (Fig. S2A). On the test set, time-dependent calibration curves and Brier scores at 3, 5, and 7 years further demonstrated good short- to mid-term discrimination and acceptable overall error (Brier scores = 0.066, 0.088, and 0.113), with generally good calibration in low-risk groups but a tendency to underestimate recurrence risk in the highest-risk subgroup, particularly at longer follow-up (Fig. S2B).

Using the MRRP model, recurrence risk scores were generated for both the training and test sets. We used the median of the training set MRRP scores as the cutoff to define high- and low-risk groups. In the test set, the high-risk group had substantially worse outcomes than the low-risk group, with an RFS hazard ratio (HR) of 6.06 (*P* < 0.001) and 5-year RFS rates of 50.0% (95% CI, 30.6 to 81.6%) versus 93.7% (95% CI, 89.3 to 98.3%), respectively (Fig. 2D). For overall survival (OS), the HR was 2.50 (*P* = 0.151), and the 5-year OS rates were 87.1% (71.8 to 100.0%) in the high-risk group and 98.2% (95.7 to 100.0%) in the low-risk group (Fig. S3A). Sensitivity analyses using alternative quantile-based thresholds (e.g., 60th/70th percentiles and tertile/quartile stratification) demonstrated similar monotonic risk gradients, with higher MRRP scores consistently associated with increased recurrence risk in the test set (Fig. S3B to E).

Further clinical subgroup analyses demonstrated consistent stratification performance of the MRRP model across subgroups, including age (≤50/>50 years), histological grade (2/3), and lymph node status (positive 1 to 3, positive ≥4, negative). Among patients with histological grade 2 tumors, who comprised more than half of the cohort, the MRRP score identified a subgroup with markedly elevated recurrence risk: In the training cohort, 5-year RFS was 77.3% in the high-risk group (*n* = 178) versus 100.0% in the low-risk group (*n* = 195; *P* < 0.001) (Fig. 2E). In the test cohort, a similar trend was observed (5-year RFS 77.8% versus 91.9% for high- versus low-risk grade 2 patients; *P* = 0.32), although the difference did not reach statistical significance because only 9 grade 2 patients were classified as high-risk (Fig. 2F). All subgroups showed significant risk stratification differences in the training set (all *P* < 0.001) (Fig. S4). Although some subgroups in the test set did not reach statistical significance (Fig. S5), possibly due to limited sample size, the risk stratification trends were consistent with those in the training cohort, indicating the model’s broad applicability.

To further assess the potential clinical utility of the MRRP score, we performed decision–curve analysis (DCA) in the test set comparing the MRRP model, a clinicopathologic model based on 7 routine variables, and “treat-all”/“treat-none” strategies at 3, 5, and 7 years. At 3 years, both models provided only limited net benefit due to the low number of early events. At 5 and 7 years, however, the MRRP model showed clearly higher net benefit than the clinicopathologic model across a broad range of clinically relevant threshold probabilities (approximately 3% to 40% and 3% to 60%, respectively) and consistently outperformed “treat-all” and “treat-none” strategies (Fig. 2G). In a subset of 68 patients with available transcriptomic data, the MRRP risk score also achieved a higher C-index than several multigene signatures approximated using the genefu package, following the procedures described in our previous work [25], including Oncotype DX, MammaPrint (GENE70), and EndoPredict (0.94 versus 0.52 to 0.60; Fig. S6), although these exploratory comparisons are limited by the small sample size.

### Ablation study of modal information fusion in MRRP

To better understand the contribution of each component in the MRRP model, we conducted stepwise ablation experiments to evaluate the distribution of prognostic information across intermodality cooperation and feature hierarchy dimensions. At the intermodality level, we observed varying impacts of different modality data on model performance. Removing the WSI module led to a significant drop in performance, with the C-index decreasing from the baseline 0.840 to 0.586 (ΔC-index = −0.254), representing nearly a 30% loss in discrimination ability. In contrast, removing the US radiomics and clinical information modules caused only minor decreases in C-index by 0.040 and 0.042, respectively (Fig. 3A). These results indicate that WSI features form the core foundation of the model’s predictive power, while US features and clinical features play auxiliary roles in optimization. To assess whether multimodal integration provides additional clinical benefit beyond discrimination metrics, we performed DCA at 3, 5, and 7 years (Fig. S7). At 3 years, net benefit was limited for all models across most thresholds, consistent with the low frequency of early recurrence. In contrast, at 5 and 7 years, MRRP maintained positive and more stable net benefit across a broader, clinically actionable range of thresholds than the ablated variants (e.g., WSI-clinical and WSI-US) and the treat-all/treat-none strategies. Although the WSI-clinical and WSI-US models may yield slightly higher net benefit at very low thresholds (near a treat-all setting), MRRP provides more consistent benefit across moderate, clinically actionable thresholds, supporting the clinical justification for multimodal integration.

To further elucidate how the MRRP model leverages these modalities, we visualized model attention using attention-score heatmaps on WSIs and gradient-weighted class activation mapping (Grad-CAM) on US regions of interest (ROIs). Based on attention scores, the MRRP model in high-risk patients primarily focuses on tumor-dense regions characterized by marked nuclear pleomorphism and a higher nuclear-to-cytoplasmic ratio; in low-risk patients, it more frequently attends to regions with milder cytologic atypia, lower tumor cellularity, and stromal areas with lymphocytic infiltration. For US, Grad-CAM was used to visualize salient lesion regions, revealing that the model mainly emphasizes signals along the tumor boundary on grayscale images. Figure S8A and B presents representative high- and low-risk patients, including WSI and US heatmaps as well as the top 20 attended WSI patches. For clinicopathologic variables, we ranked variable importance using attention weights, and the results show that Ki-67, histologic grade, lymph node status, tumor size, and HER2 are the top 5 features emphasized by the model (Fig. S9). Together, these findings support the biological and clinical plausibility of the learned multimodal patterns.

Intramodality ablation experiments revealed differing contributions of internal features to model performance. In the WSI modality, absence of deep features resulted in a significant performance decline (C-index decreased by 0.256 and 0.198), far exceeding the impact of missing morphological (ΔC-index = −0.021) or topological features (ΔC-index = −0.032) (Fig. 3B). When using single WSI features, the deep feature model (C-index = 0.796) outperformed both morphological and topological feature models (both 0.505). However, the single deep feature model performed worse than multi-feature combinations with morphological features (C-index: 0.808) or topological feature (C-index: 0.819) (Fig. 3B). These findings suggest that deep features contribute most significantly within the WSI modality, while morphological and topological features synergistically enhance performance. Moreover, in WSI unimodal analysis, the deep feature model (C-index: 0.741) also surpassed topological feature (0.586) and morphological feature (0.483) models (Fig. S10A).

In the US modality, removing radiomics features caused a notable performance drop (ΔC-index = −0.209), whereas missing deep features (ΔC-index = −0.024 and −0.052) and text embeddings (ΔC-index = −0.006) had relatively minor effects (Fig. 3C). For single US features, the radiomics model (C-index = 0.826) outperformed deep features (0.685) and text embedding models (0.785). The single radiomics model’s performance was slightly inferior only to its combination with deep features (C-index: 0.834) (Fig. 3C). Interestingly, in US unimodal analysis, the deep feature model (C-index = 0.620) showed the best performance, surpassing text embeddings (0.529) and radiomics (0.482) (Fig. S10B). This contrasts with multimodal results, indicating complex intermodality interactions.

Comparing intermodality fusion strategies, the MRRP’s cross-attention mechanism demonstrated clear superiority, achieving a C-index of 0.840, which is 3% to 7% higher than traditional feature concatenation (0.803), addition (0.770), and multiplication (0.757) methods (Fig. 3D). This advantage stems from its dynamic weight allocation capability, effectively capturing nonlinear associations across data sources. These results highlight MRRP’s effective integration of multimodal information.

### Validation of the effectiveness of intramodality interaction mechanism

To investigate the impact of query vector (Query) selection in the intramodality cross-attention mechanism on model performance, we conducted a series of feature interaction experiments (Table 1). In the WSI unimodal model, using deep features as the Query yielded the best performance (C-index = 0.757), while selecting morphological or topological features as Query led to decreases of 36.3% (C-index = 0.482, Δ = −0.275) and 20.2% (C-index = 0.604, Δ = −0.153), respectively. A similar trend was observed in the US unimodal model, where using text embeddings as Query resulted in a significantly higher C-index (0.561) compared to other choices, improving by 19.4% and 14.2% over image deep features (0.470) and radiomics features (0.491), respectively. When extending the feature interaction experiments to multimodal combinations, an interesting phenomenon emerged: the optimal Query strategy in unimodal settings did not always hold in multimodal contexts. Analysis of the full MRRP framework showed that the combination of WSI deep features and US deep features as Query (C-index = 0.840) outperformed the unimodal best combination (WSI deep feature + US text embeddings, C-index = 0.817). This finding suggests the existence of complex synergistic effects in multimodal interactions. Further analysis revealed that in the WSI-clinical model, deep features retained their dominant role. In contrast, in the US-clinical model, deep features replaced the unimodal optimal text embeddings as the more effective Query choice (Table S2).

**Table 1.** Model performance with different query vectors in intramodality cross-attention mechanism

Modal	WSI features (Q)			US features (Q)			Clinical	C-index (Δ)
	Deep	Morph	Top	Deep	Omic	Text		
WSI	✓							0.757 (Ref)
		✓						0.482 (−0.275)
			✓					0.604 (−0.153)
US				✓				0.470 (−0.091)
					✓			0.491 (−0.070)
						✓		0.561 (Ref)
MRRP	✓			✓			✓	0.840 (Ref)
		✓		✓			✓	0.553 (−0.287)
			✓	✓			✓	0.604 (−0.236)
	✓				✓		✓	0.801 (−0.039)
	✓					✓	✓	0.817 (−0.023)

Deep, deep learning-based image features extracted from WSIs/US; Morph, handcrafted morphological features; Top, graph-based spatial topological features derived from cell–cell relationships; Omic, quantitative radiomics features extracted from US tumor regions using the PyRadiomics library; Text, features derived from text US diagnostic reports

### Selection of pretrained encoders in the MRRP model

With the rapid development of medical AI, pretrained encoders are continuously evolving. Selecting appropriate encoders has become a key factor influencing model performance. We systematically evaluated the performance of mainstream pretrained encoders according to different feature extraction requirements. In the WSI unimodal model, for extracting WSI deep features, the UNI (a general-purpose self-supervised vision encoder for histopathology) encoder outperformed other mainstream encoders such as CONCH (a vision-language foundation model for computational pathology) and REMEDIS (a large-scale self-supervised medical imaging foundation model) with a C-index of 0.757, benefiting from its pretraining on millions of pathology images (Fig. 4A). Similarly, in the US unimodal model, the BioGPT large model, designed specifically for the biomedical domain, excelled in extracting US text features (C-index = 0.547), surpassing other large models (Fig. 4A). Interestingly, in the multimodal model, although UNI and BioGPT were the best performers in their respective unimodal tasks, the combination of UNI and the general-domain BERT model produced the best synergy within the full MRRP framework (C-index = 0.840). Further analysis showed that UNI maintained its advantage in the WSI-clinical model (Fig. S11A), whereas BERT outperformed the unimodal best BioGPT in the US-clinical model (Fig. S11B). These findings suggest that in multimodal modeling, the choice of pretrained encoders cannot simply follow the unimodal best solution; the complex interactions among features from different modalities must also be considered.

### Multimodal fusion with missing data

In real clinical settings, multimodal patient data often suffer from partial missingness. To address this common challenge, we extended the MRRP framework by developing a learnable compensation mechanism. Given that clinical information is the most readily available data in medical practice, we specifically optimized compensation strategies for scenarios with missing WSI or US modalities. As shown in Table 2, under the WSI missing scenario, the model with learnable compensation (C-index = 0.641) outperformed the baseline method that simply ignored the missing modality (C-index = 0.586). Its predictive performance was between the complete modality model (C-index = 0.840) and the completely missing scenario (C-index = 0.586). A similar compensation effect was observed when the US modality was missing: The compensated model (C-index = 0.820) performed between the full modality model (C-index = 0.840) and the missing modality model (C-index = 0.800). Our proposed learnable compensation method effectively mitigates the common issue of modality missingness in clinical practice, enhancing the applicability of the MRRP model in real-world medical scenarios.

**Table 2.** Performance of the missing modality compensation mechanism models

odel	WSI features		US features		Clinical	C-index
	Available	Learnable	Available	Learnable		
Missing WSI			✓		✓	0.586
		✓	✓		✓	0.641
Missing US	✓				✓	0.800
	✓			✓	✓	0.820
MRRP	✓		✓		✓	0.840

## Discussion

Patients with HR^+^/HER2^−^ EBC face a long-term risk of recurrence. Even after completing 5 years of standard endocrine therapy with or without adjuvant chemotherapy, the risk of disease relapse and death persists [2,26–29]. Although many prognostic tools have been developed and some are applied clinically [30–34], their predictive performance remains limited in certain scenarios [35].

In this study, we successfully developed the MRRP by integrating routinely available clinical data, including pathology images, US grayscale images, US text reports, and structured clinical parameters. The model showed good predictive performance on the test set, with a C-index of 0.840, significantly outperforming any single-modality model by 11% to 79%. Notably, MRRP maintained stable performance in long-term prediction, with a 5-year follow-up AUC of 0.900, making it particularly suitable for guiding long-term clinical follow-up decisions. Compared to the CIMPTGV model developed by Zhang’s team [21] (C-index = 0.869), which integrates multi-omics data including genomics, transcriptomics, and metabolomics, among others, MRRP’s absolute predictive accuracy is slightly lower. However, MRRP uses data routinely collected in clinical practice without requiring extra specialized tests or costs. In addition, while the training set median was used as a prespecified cutoff for defining high- versus low-risk groups and sensitivity analyses with alternative quantile-based thresholds showed consistent risk gradients, the optimal clinical thresholds for actionable decision-making will need to be calibrated prospectively and may vary across institutions and patient populations.

Our ablation experiments revealed the differential contributions of multimodal data in prognostic prediction. Particularly, removing pathological features led to a significant 30% drop in model performance, confirming the central role of pathology image features in breast cancer prognosis. This finding aligns with previous reports on the prognostic value of pathology images in gastric cancer [36], colorectal cancer [37], and ovarian cancer [15], underscoring the general importance of pathology image analysis in tumor prognosis. Similarly, recent AI-based models using routine H&E-stained WSIs in breast cancer, such as DeepGrade and the CE-IVD-marked Stratipath Breast solution, have demonstrated independent prognostic value and, in the case of Stratipath, substantial agreement with an established multigene assay in ER^+^/HER2^−^ EBC [10–12]. By contrast, the US-only model achieved a C-index of 0.470, indicating limited discriminative ability when US is used alone, although US features provided modest incremental benefit when integrated with WSI and clinical variables in the MRRP framework. This limited standalone performance likely reflects the subtle differences in preoperative grayscale US appearance among HR^+^/HER2^−^ EBC, the reliance on a single 2D grayscale image per patient without additional functional information (e.g., doppler or elastography), and the inherent operator and machine dependence of US despite standardized image selection and ROI annotation. Future work will explore incorporating richer multiparametric US data and more standardized acquisition protocols to enhance the contribution of US to multimodal risk prediction.

Methodologically, unlike most prior studies relying on single feature types (deep or morphological features), we systematically integrated 3 complementary pathological feature representations: deep features, morphological features, and topological features. This multidimensional fusion strategy is still rare in related fields, with few reports such as in HER2-targeted gastric cancer treatment response prediction [36]. Our results show that this combination effectively captures complex prognostic patterns that single feature types cannot detect, offering a novel approach for future pathology-based prognostic research. We also found that although US features alone have limited predictive power, their combination with other features improved model performance by 5%, highlighting the complementary value of multimodal data.

During model development, feature interaction experiments revealed that the optimal query selection strategy in unimodal settings does not necessarily translate to multimodal scenarios. For example, the best unimodal query combination (WSI deep + US text) was not the best in multimodal modeling. This likely reflects complex synergy effects among features influencing query selection. Similarly, pretrained encoder choices that were optimal unimodally did not always yield the best multimodal combination. These findings suggest that multimodal model design should not simply replicate unimodal optimization but require systematic experimentation to find the best combinations, providing theoretical and practical guidance for future multimodal predictive models.

To address common clinical challenges of missing modality data, we developed a learnable compensation strategy. Experiments confirmed that this approach effectively improved model performance under missing data: Compensation increased performance by 9.4% when WSI modality was missing and by 2.5% when US modality was missing. This enhancement improves MRRP’s applicability in real-world clinical settings. Our compensation strategy is conceptually similar to the method used by Chen’s team [36] in their HER2-targeted therapy prediction model for gastric cancer, as both rely on learnable parameters to compensate for missing modality features. In contrast, Can Cui et al. [38] proposed a different multimodal learning pipeline (MMD). These studies collectively advance multimodal medical AI toward clinical utility by addressing data incompleteness. Future work may explore adaptive compensation mechanisms for more complex missing data scenarios.

Despite promising results, this study has several limitations. First, it is a single-center retrospective analysis with an internal train-test split and relatively few recurrence events in the test set. Although we performed extensive internal validation, these procedures cannot substitute for true external validation. Multicenter prospective or large independent cohorts will be needed to confirm the generalizability of the MRRP model. Second, although all patients received postoperative chemo-endocrine therapy (C-ET), detailed treatment information (regimens, radiotherapy, and endocrine duration/adherence) was not consistently available and thus not modeled, so residual treatment-related confounding cannot be excluded. Future prospective or multicenter cohorts with comprehensive treatment documentation are needed for adjustment and validation. Third, the current MRRP implementation relies on manual tumor annotation on both WSIs and US images by specialists and therefore functions as a semi-automated decision-support tool rather than a fully automated system; integrating robust automatic segmentation and ROI detection will be important for future workflow integration. Finally, emerging molecular biomarkers were not incorporated, and future versions should assess whether selected markers provide additional prognostic value beyond routine clinicopathologic data.

In conclusion, we successfully developed and validated the MRRP model, a multimodal risk prediction tool for recurrence in HR^+^/HER2^−^ EBC, leveraging routinely collected clinical data including pathology, US, and clinical parameters. This model underscores the value of routine multimodal data for long-term recurrence prediction, highlights the pivotal role of pathology features, and uncovers unique patterns of multimodal interaction. MRRP demonstrates the promise of integrating AI with routinely available clinical data, offering broad applicability due to its reliance on accessible information. With further multicenter validation and dynamic optimization, this approach holds potential to enhance personalized follow-up and treatment decision-making for patients with HR^+^/HER2^−^ EBC.

## Materials and Methods

### Data collection

This study included 768 patients with HR^+^/HER2^−^ EBC who were recruited at WCH, Sichuan University, between January 2005 and December 2017. Eligibility criteria for the WCH cohort followed our previously published study on recurrence risk prediction in HR^+^/HER2^−^ EBC [39]. Specifically, the inclusion criteria were as follows: (a) unilateral primary invasive breast cancer, clinically staged as I to III at the time of initial diagnosis; (b) receipt of adjuvant C-ET within 3 months after surgery, without any preoperative systemic treatment; and (c) availability of a definitive postoperative pathological diagnosis. Patients were excluded if they (a) had clinical stage IV disease; (b) had multifocal or bilateral invasive breast cancer; (c) received only adjuvant endocrine therapy or only chemotherapy; or (d) had incomplete clinicopathologic, imaging, or follow-up information. All included patients underwent postoperative adjuvant C-ET according to institutional protocols and contemporary guidelines. However, detailed information on specific chemotherapy regimens, radiotherapy fields/doses, and endocrine therapy duration or adherence was incompletely and inconsistently recorded over the study period, and therefore, these treatment variables were not incorporated as structured covariates in the current analysis. All data were collected by experienced physicians. Pathologists selected representative pathology slides, US specialists selected representative grayscale US images, and clinical physicians recorded clinical information and follow-up outcomes. Follow-up was conducted every 3 to 4 months during the first 2 years after diagnosis, every 6 months from the third to the fifth year, and once a year thereafter. RFS was defined as the time from surgery to local recurrence, distant metastasis, or disease-related death. OS was defined as the time from surgery to death from any cause.

### Pathology slide preprocessing and feature extraction

Because the patient enrollment period in this study spanned more than a decade (2005–2017), we first collected all routine H&E-stained slides for enrolled patients in the WCH cohort and performed systematic microscopic quality assessment. A substantial proportion of early slides did not meet the image quality requirements for subsequent digital pathology analysis (e.g., fading, uneven staining, and tissue folds). Therefore, we retrieved the corresponding formalin-fixed, paraffin-embedded (FFPE) tissue blocks for all eligible patients, resectioned them at 4-μm thickness, and restained them with H&E using the same automated stainer. The newly prepared H&E slides were digitized using a UNIC scanner at 40× optical magnification (0.25 μm/pixel resolution) to generate high-quality WSIs. Because all WSIs were produced under this unified cutting, staining, and scanning protocol, we did not perform additional color normalization. To ensure accurate identification of tumor regions, all WSIs were manually annotated by mid-level pathologists using ASAP software version 1.8 (https://github.com/computationalpathologygroup/ASAP/releases) who were blinded to patient outcomes and to US or other modality-specific information.

We extracted multidimensional features from the pathology images, including deep features, handcrafted morphological features, and spatial topological features. For deep feature extraction, we used pretrained deep learning encoders such as CONCH [40] and UNI [41], among others, which were trained on large-scale pathology datasets through self-supervised learning. These encoders are capable of capturing complex histological patterns in WSIs and offer strong feature representation and transfer learning capabilities. Each WSI was divided into image patches, which were then fed into the encoders to generate corresponding deep feature vectors as the base representations.

For handcrafted features, we adopted sc-MTOP framework [23], in which a Hover-Net model [42] pretrained on the public PanNuke dataset is used to perform nuclear segmentation and cell-type classification (tumor, inflammatory, stromal, and normal nuclei) on WSIs. Based on these results, sc-MTOP then computed handcrafted features: (a) morphological features, including geometric attributes of individual cells (area, perimeter, bounding box area, major/minor axis length, eccentricity, and circularity), curvature features (mean, maximum, and minimum curvature), texture statistics derived from the gray-level co-occurrence matrix (GLCM) such as angular second moment (ASM), contrast, entropy, and homogeneity, as well as mean and standard deviation of intensity within cell regions, and (b) spatial topological features, where a *k*-nearest neighbor (*k* = 5) graph was constructed using the spatial locations of cells. From this graph, we extracted multi-dimensional graph-theoretic metrics, including node-level features (degree, nucleus count, clustering coefficient), centrality features (closeness centrality, betweenness centrality), and spatial distance features (minimum and average adjacent edge length, in micrometers). Detailed feature definitions are provided in Table S3.

### US grayscale image preprocessing and feature extraction

For US grayscale image analysis, we adopted a systematic preprocessing and feature extraction pipeline. For each patient, we used the last preoperative grayscale US examination performed within 2 weeks before surgery as the index study. Because this retrospective cohort spanned a long accrual period, US examinations were performed using multiple mainstream scanners, including Philips iU22, Philips HD11, Philips HDI 5000, Siemens Acuson Sequoia 512, and GE LOGIQ 9. Accordingly, variability arising from inter-scanner differences and operator-dependent factors (e.g., patient-specific parameter tuning, scanning technique, and plane selection) is unavoidable in long-term retrospective data. To mitigate such variability at the data curation stage, all images were derived from standardized breast US examinations for breast cancer diagnosis, performed by professionally trained operators following departmental scanning protocols to ensure acquisition of key lesion-relevant views. We asked experienced breast US specialists to review all examinations and, for each patient, select the clearest and most representative tumor image according to departmental practice standards. Then, experienced breast US specialists annotated all selected grayscale images using the LabelImg tool [Tzutalin. LabelImg. Git code (2015). https://github.com/tzutalin/labelImg] while being blinded to recurrence outcomes and to pathology/WSI-derived features. Tumor regions were precisely localized with rectangular bounding boxes, and all patient identifiers were removed for anonymization. Based on the annotations, we extracted the tumor ROI with a 6-pixel margin around the bounding box to preserve potential biological features at the tumor boundary.

For deep feature extraction, we trained a lightweight encoder based on the ResNet architecture. The model received ROI images resized to 256 × 256 pixels as input and produced feature maps from the last residual block (dimensions: 64 × 16 × 16). These deep features captured high-level visual patterns of the tumor.

For radiomics analysis, we used the open-source PyRadiomics library (v3.0.1) to extract 4 categories of quantitative features. First-order statistical features, such as energy, entropy, mean intensity, standard deviation, kurtosis, and skewness, described the distribution of pixel intensities. Texture features, derived from the GLCM, gray-level run-length matrix (GLRLM), and gray-level size zone matrix (GLSZM), quantified image patterns such as contrast, correlation, and homogeneity. Two-dimensional shape features, such as maximum and minimum diameter and pixel area, characterized the tumor geometry. High-order texture features were obtained through multi-scale image transformations, including wavelet decomposition and Laplacian of Gaussian filtering. This multi-level feature extraction strategy ensured a comprehensive characterization of tumor imaging phenotypes.

### US diagnostic report preprocessing and feature extraction

We used regular expressions to accurately extract text related to breast US from the original diagnostic reports. All extracted content was reviewed by clinical physicians. Given the linguistic characteristics of Chinese medical text, we employed the open-source biomedical large language model openBioLLM-70B [43] to translate the Chinese reports into English. A team of physicians then verified semantic consistency to ensure that key clinical information was preserved during language conversion.

For feature extraction, we explored several advanced text representation methods. For the English reports, we evaluated domain-specific biomedical models, including CONCH [40], BioGPT [44], and BiomedBERT [45], and also tested the performance of the general-domain BERT-large-uncased model [46]. For the normalized Chinese reports, we applied the BERT-base-Chinese model [46] to generate features. These pretrained language models can capture complex medical semantic embeddings in the US reports, including lesion morphology and hemodynamic characteristics, enabling the construction of high-quality text feature representations that comprehensively reflect tumor characteristics.

### Clinical information representation

The clinical information collected in this study included patient age, tumor size, lymph node status, histological grade, ER, progesterone receptor (PR), HER2, and Ki67 status, as well as molecular subtype. To fully leverage the predictive value of these structured data, we designed a dedicated feature encoding strategy. Continuous variables, such as age and tumor size, were mapped to a 1,024-dimensional feature space using a learnable linear transformation layer (nn.Linear). Categorical variables, such as molecular subtype and lymph node status, were embedded into a 1,024-dimensional space using a high-dimensional embedding layer (nn.Embedding) to learn their semantic representations. All encoded continuous and categorical features were then concatenated along the feature dimension to form a unified clinical feature representation, which served as the structured input for subsequent multimodal interactions. This approach preserved the clinical meaning of the original variables while enabling optimized feature representations through deep learning.

### Multimodal information fusion in the MRRP model

The MRRP model developed in this study is based on a transformer architecture and is designed to integrate multi-source data for accurate recurrence risk prediction in HR^+^/HER2^−^ EBC. The model employs a hierarchical fusion strategy consisting of 3 modules: intramodality fusion, intermodality fusion, and integration of patient clinical information (Fig. 1B-1 to B-4).

In the intramodality fusion module, we deeply integrated features within the pathology and US modalities. For pathology, 3 complementary feature representations were combined: deep features (F_WSI-deep_), morphological features (F_WSI-morph_), and topological features (F_WSI-top_). Using a novel cross-attention mechanism, we first used Fwsi-deep as the query vector and F_WSI-top_ as the key–value pair to learn deep features enriched with spatial contextual information. In parallel, F_WSI-deep_ was used as the query vector and F_WSI-morph_ as the key–value pair to construct features constrained by morphological information. The reconstructed features were then element-wise summed with the original Fwsi-deep to generate the integrated pathomics representation (F_Path_).

For US, we combined image deep features (F_US-deep_), quantitative radiomics features (F_US-omic_), and textual semantic features (F_US-text_). Following a similar strategy, F_US-deep_ served as the query baseline for cross-attention with F_US-omic_ and F_US-text_. The former enhanced the semantic expressiveness of radiomics features, while the latter established a mapping between imaging and text. The fused outputs were aggregated to form the unified US representation (F_US_). This symmetric fusion design ensured organic integration of features within each modality and prepared them for subsequent cross-modality interactions.

In the intermodality fusion stage, F_Path_ and F_US_ were first dimension-aligned through a fully connected layer and then concatenated to form cross-modality fusion features (F_Inter_). In the patient-level fusion module, a cross-attention mechanism was applied for deeper interaction: F_Inter_ was used as the query vector, and clinical features served as key–value pairs, allowing dynamic integration of multi-source information via attention weights. The cross-attention outputs were connected to the original Finter through a residual connection (element-wise addition), preserving essential information from the original cross-modality features while incorporating clinically guided insights. The resulting representation provided a comprehensive characterization of each patient’s disease profile. This progressive hierarchical fusion architecture, supported by a feature retention mechanism, fully exploited cross-modality synergies while preserving the domain-specific characteristics of each modality.

### Experimental design

The MRRP model was trained using a 2-stage strategy. In the first stage, modality-specific features were extracted using dedicated pretrained encoders for pathomics and US radiomics. In the second stage, the extracted pathology and US features, together with learnable clinical feature representations, were fed into the multimodal fusion network, and the entire architecture was then optimized in an end-to-end manner. This staged training paradigm preserved the advantages of modality-specific feature representations while enabling coordinated optimization of cross-modality interactions. Model optimization was performed using the Cox proportional hazards partial likelihood loss function [47], which is well suited for handling right-censored survival data. During training, we applied several regularization strategies to reduce overfitting. A dropout rate of 0.2 was used in the intermodal fusion module to reduce co-adaptation among neurons, all cross-attention layers were implemented with a single attention head to limit model complexity and the number of trainable parameters, and the model was optimized using the Adam optimizer with weight decay (4 × 10^−6^), introducing a mild L2 regularization effect to constrain parameter magnitudes.

Based on the WCH cohort of HR^+^/HER2^−^ EBC patients (*n* = 768), the dataset was divided into a training set (*n* = 640) and a test set (*n* = 128) using stratified random sampling to ensure balanced distribution of samples. Model performance was objectively assessed using the C-index, which quantifies the agreement between the predicted risk ranking and the actual observed outcomes.

### Handling missing modality inputs in intermodal fusion

When a modality-specific representation required by intermodal fusion is unavailable, e.g., the WSI-derived feature embedding is missing for a given patient, we replace the absent modality input with a learnable embedding vector. Concretely, we instantiate a parameter missing embedding using torch.nn.Parameter, initialized to zeros and matched in dimensionality to the corresponding modality feature. For each sample, a binary availability indicator (mask) is used to determine whether the modality feature is observed. During the forward pass, the missing modality slot in the fusion input is filled with missing embedding, whereas observed modalities retain their original embeddings. The completed multimodal input is then fed into the intermodal fusion backbone to produce the fused representation and final prediction.

Training is performed end-to-end under the standard supervised objective. The loss is computed using ground-truth labels, and gradients backpropagate through the fusion backbone into missing embedding, allowing it to be updated jointly with all other network parameters by the optimizer. Through iterative optimization, missing embedding adaptively learns a data-driven representation that compensates for the missing modality input, enabling robust fusion and inference under incomplete multimodal inputs.

### Statistical analysis

All statistical analyses and visualizations were performed using R (version 4.3.2). Kaplan–Meier curves and the log-rank test were used to assess differences in RFS and OS between high-risk and low-risk groups. Time-dependent AUC was plotted using the R packages “riskRegression” and “survival”. Differences in variables between groups were compared using the Wilcoxon test. Spearman’s rank correlation was used to evaluate associations between the MRRP score and various variables. Model performance was assessed using the C-index and time-dependent AUC. A 2-sided *P* value of <0.05 was considered statistically significant.

## Data Availability

The in-house data used in this study are available from the corresponding author upon reasonable request and with a signed data access agreement. De-identified patient-level clinical data will be provided upon reasonable request. The source code of the deep learning model developed in this study is publicly available online (https://github.com/77-kate/mrrp).

## References

[1] Huppert LA, Gumusay O, Idossa D, Rugo HS. Systemic therapy for hormone receptor-positive/human epidermal growth factor receptor 2-negative early stage and metastatic breast cancer. CA Cancer J Clin. 2023;73(5):480–515. 36939293 10.3322/caac.21777

[2] Pan H, Gray R, Braybrooke J, Davies C, Taylor C, McGale P, Peto R, Pritchard KI, Bergh J, Dowsett M, et al. 20-year risks of breast-cancer recurrence after stopping endocrine therapy at 5 years. N Engl J Med. 2017;377(19):1836–1846. 29117498 10.1056/NEJMoa1701830 PMC5734609

[3] Goldstein LJ, Gray R, Badve S, Childs BH, Yoshizawa C, Rowley S, Shak S, Baehner FL, Ravdin PM, Davidson NE, et al. Prognostic utility of the 21-gene assay in hormone receptor-positive operable breast cancer compared with classical clinicopathologic features. J Clin Oncol. 2008;26(25):4063–4071. 18678838 10.1200/JCO.2007.14.4501 PMC2654377

[4] Sparano JA, Gray RJ, Makower DF, Pritchard KI, Albain KS, Hayes DF, Geyer CE Jr, Dees EC, Goetz MP, Olson JA Jr, et al. Adjuvant chemotherapy guided by a 21-gene expression assay in breast cancer. N Engl J Med. 2018;379(2):111–121. 29860917 10.1056/NEJMoa1804710 PMC6172658

[5] Weiser R, Haque W, Polychronopoulou E, Hatch SS, Kuo YF, Gradishar WJ, Klimberg VS. The 21-gene recurrence score in node-positive, hormone receptor-positive, HER2-negative breast cancer: A cautionary tale from an NCDB analysis. Breast Cancer Res Treat. 2021;185(3):667–676. 33070279 10.1007/s10549-020-05971-1

[6] Mook S, Schmidt MK, Viale G, Pruneri G, Eekhout I, Floore A, Glas AM, Bogaerts J, Cardoso F, Piccart-Gebhart MJ, et al. The 70-gene prognosis-signature predicts disease outcome in breast cancer patients with 1-3 positive lymph nodes in an independent validation study. Breast Cancer Res Treat. 2009;116(2):295–302. 18661261 10.1007/s10549-008-0130-2

[7] Tsai M, Lo S, Audeh W, Qamar R, Budway R, Levine E, Whitworth P, Mavromatis B, Zon R, Oldham D, et al. Association of 70-gene signature assay findings with physicians’ treatment guidance for patients with early breast cancer classified as intermediate risk by the 21-gene assay. JAMA Oncol. 2018;4(1): Article e173470. 29075751 10.1001/jamaoncol.2017.3470 PMC5833645

[8] Bueno-de-Mesquita JM, Linn SC, Keijzer R, Wesseling J, Nuyten DS, van Krimpen C, Meijers C, de Graaf PW, Bos MM, Hart AA, et al. Validation of 70-gene prognosis signature in node-negative breast cancer. Breast Cancer Res Treat. 2009;117(3):483–495. 18819002 10.1007/s10549-008-0191-2

[9] Martin M, Brase JC, Ruiz A, Prat A, Kronenwett R, Calvo L, Petry C, Bernard PS, Ruiz-Borrego M, Weber KE, et al. Prognostic ability of EndoPredict compared to research-based versions of the PAM50 risk of recurrence (ROR) scores in node-positive, estrogen receptor-positive, and HER2-negative breast cancer. A GEICAM/9906 sub-study. Breast Cancer Res Treat. 2016;156(1):81–89. 26909792 10.1007/s10549-016-3725-z PMC4788691

[10] Wang Y, Acs B, Robertson S, Liu B, Solorzano L, Wählby C, Hartman J, Rantalainen M. Improved breast cancer histological grading using deep learning. Ann Oncol. 2022;33(1):89–98. 34756513 10.1016/j.annonc.2021.09.007

[11] Sharma A, Lövgren SK, Eriksson KL, Wang Y, Robertson S, Hartman J, Rantalainen M. Validation of an AI-based solution for breast cancer risk stratification using routine digital histopathology images. Breast Cancer Res. 2024;26(1):123. 39143539 10.1186/s13058-024-01879-6 PMC11323658

[12] Wang Y, Sun W, Karlsson E, Kang Lövgren S, Ács B, Rantalainen M, Robertson S, Hartman J. Clinical evaluation of deep learning-based risk profiling in breast cancer histopathology and comparison to an established multigene assay. Breast Cancer Res Treat. 2024;206(1):163–175. 38592541 10.1007/s10549-024-07303-z PMC11182789

[13] Yu KH, Beam AL, Kohane IS. Artificial intelligence in healthcare. Nat Biomed Eng. 2018;2(10):719–731. 31015651 10.1038/s41551-018-0305-z

[14] He J, Baxter SL, Xu J, Xu J, Zhou X, Zhang K. The practical implementation of artificial intelligence technologies in medicine. Nat Med. 2019;25(1):30–36. 30617336 10.1038/s41591-018-0307-0 PMC6995276

[15] Boehm KM, Aherne EA, Ellenson L, Nikolovski I, Alghamdi M, Vázquez-García I, Zamarin D, Long Roche K, Liu Y, Patel D, et al. Multimodal data integration using machine learning improves risk stratification of high-grade serous ovarian cancer. Nat Cancer. 2022;3(6):723–733. 35764743 10.1038/s43018-022-00388-9 PMC9239907

[16] Lipkova J, Chen RJ, Chen B, Lu MY, Barbieri M, Shao D, Vaidya AJ, Chen C, Zhuang L, Williamson DFK, et al. Artificial intelligence for multimodal data integration in oncology. Cancer Cell. 2022;40(10):1095–1110. 36220072 10.1016/j.ccell.2022.09.012 PMC10655164

[17] Hao J, Kosaraju SC, Tsaku NZ, Song DH, Kang M. PAGE-Net: Interpretable and integrative deep learning for survival analysis using histopathological images and genomic data. Pac Symp Biocomput. 2020;25:355–366. 31797610

[18] Ning Z, Xiao Q, Feng Q, Chen W, Zhang Y. Relation-induced multi-modal shared representation learning for Alzheimer’s disease diagnosis. IEEE Trans Med Imaging. 2021;40(6):1632–1645. 33651685 10.1109/TMI.2021.3063150

[19] Chen RJ, Lu MY, Wang J, Williamson DFK, Rodig SJ, Lindeman NI, Mahmood F. Pathomic fusion: An integrated framework for fusing histopathology and genomic features for cancer diagnosis and prognosis. IEEE Trans Med Imaging. 2022;41(4):757–770. 32881682 10.1109/TMI.2020.3021387 PMC10339462

[20] Hu C, Chen W, Li F, Zhang Y, Yu P, Yang L, Huang L, Sun J, Chen S, Shi C, et al. Deep learning radio-clinical signatures for predicting neoadjuvant chemotherapy response and prognosis from pretreatment CT images of locally advanced gastric cancer patients. Int J Surg. 2023;109(7):1980–1992. 37132183 10.1097/JS9.0000000000000432 PMC10389454

[21] Zhang H, Yang F, Xu Y, Zhao S, Jiang YZ, Shao ZM, Xiao Y. Multimodal integration using a machine learning approach facilitates risk stratification in HR+/HER2- breast cancer. Cell Rep Med. 2025;6(2): Article 101924. 39848244 10.1016/j.xcrm.2024.101924 PMC11866502

[22] Cheerla A, Gevaert O. Deep learning with multimodal representation for pancancer prognosis prediction. Bioinformatics. 2019;35(14):i446–i454. 31510656 10.1093/bioinformatics/btz342 PMC6612862

[23] Zhao S, Chen DP, Fu T, Yang JC, Ma D, Zhu XZ, Wang XX, Jiao YP, Jin X, Xiao Y, et al. Single-cell morphological and topological atlas reveals the ecosystem diversity of human breast cancer. Nat Commun. 2023;14(1):6796. 37880211 10.1038/s41467-023-42504-y PMC10600153

[24] van Griethuysen JJM, Fedorov A, Parmar C, Hosny A, Aucoin N, Narayan V, Beets-Tan RGH, Fillion-Robin JC, Pieper S, Aerts HJWL. Computational radiomics system to decode the radiographic phenotype. Cancer Res. 2017;77(21):e104–e107. 29092951 10.1158/0008-5472.CAN-17-0339 PMC5672828

[25] Wu X, Lu X, Zhang W, Zhong X, Bu H, Zhang Z. Development and validation of a 10-gene signature for predicting recurrence risk in HR+/HER2- early breast cancer undergoing chemo-endocrine therapy. Breast. 2025;82: Article 104484. 40288025 10.1016/j.breast.2025.104484 PMC12056407

[26] Colleoni M, Sun Z, Price KN, Karlsson P, Forbes JF, Thürlimann B, Gianni L, Castiglione M, Gelber RD, Coates AS, et al. Annual hazard rates of recurrence for breast cancer during 24 years of follow-up: Results from the international breast cancer study group trials I to V. J Clin Oncol. 2016;34(9):927–935. 10.1200/JCO.2015.62.3504 PMC4933127 26786933

[27] Regan MM, Neven P, Giobbie-Hurder A, Goldhirsch A, Ejlertsen B, Mauriac L, Forbes JF, Smith I, Láng I, Wardley A, et al. Assessment of letrozole and tamoxifen alone and in sequence for postmenopausal women with steroid hormone receptor-positive breast cancer: The BIG 1-98 randomised clinical trial at 8.1 years median follow-up. Lancet Oncol. 2011;12(12):1101–1108. 22018631 10.1016/S1470-2045(11)70270-4 PMC3235950

[28] Slamon DJ, Fasching PA, Hurvitz S, Chia S, Crown J, Martín M, Barrios CH, Bardia A, Im SA, Yardley DA, et al. Rationale and trial design of NATALEE: A phase III trial of adjuvant ribociclib + endocrine therapy versus endocrine therapy alone in patients with HR+/HER2- early breast cancer. Ther Adv Med Oncol. 2023;15:17588359231178125. 37275963 10.1177/17588359231178125 PMC10233570

[29] Early Breast Cancer Trialists’ Collaborative Group. Relevance of breast cancer hormone receptors and other factors to the efficacy of adjuvant tamoxifen: Patient-level meta-analysis of randomised trials. Lancet. 2011;378(9793):771–784. 21802721 10.1016/S0140-6736(11)60993-8 PMC3163848

[30] Paik S, Shak S, Tang G, Kim C, Baker J, Cronin M, Baehner FL, Walker MG, Watson D, Park T, et al. A multigene assay to predict recurrence of tamoxifen-treated, node-negative breast cancer. N Engl J Med. 2004;351(27):2817–2826. 15591335 10.1056/NEJMoa041588

[31] Van’t Veer LJ, Dai H, Van De Vijver MJ, He YD, Hart AA, Mao M, Peterse HL, Van Der Kooy K, Marton MJ, Witteveen AT, et al. Gene expression profiling predicts clinical outcome of breast cancer. Nature. 2002;415(6871):530–536. 11823860 10.1038/415530a

[32] Sotiriou C, Wirapati P, Loi S, Harris A, Fox S, Smeds J, Nordgren H, Farmer P, Praz V, Haibe-Kains B, et al. Gene expression profiling in breast cancer: Understanding the molecular basis of histologic grade to improve prognosis. J Natl Cancer Inst. 2006;98(4):262–272. 16478745 10.1093/jnci/djj052

[33] Filipits M, Rudas M, Jakesz R, Dubsky P, Fitzal F, Singer CF, Dietze O, Greil R, Jelen A, Sevelda P, et al. A new molecular predictor of distant recurrence in ER-positive, HER2-negative breast cancer adds independent information to conventional clinical risk factors. Clin Cancer Res. 2011;17(18):6012–6020. 21807638 10.1158/1078-0432.CCR-11-0926

[34] Parker JS, Mullins M, Cheang MC, Leung S, Voduc D, Vickery T, Davies S, Fauron C, He X, Hu Z, et al. Supervised risk predictor of breast cancer based on intrinsic subtypes. J Clin Oncol. 2023;41(8):4192–4199. 37672882 10.1200/JCO.22.02511

[35] Prat A, Parker JS, Fan C, Cheang MCU, Miller LD, Bergh J, Chia SKL, Bernard PS, Nielsen TO, Ellis MJ, et al. Concordance among gene expression-based predictors for ER-positive breast cancer treated with adjuvant tamoxifen. Ann Oncol. 2012;23(11):2866–2873. 22532584 10.1093/annonc/mds080 PMC3477878

[36] Chen Z, Chen Y, Sun Y, Tang L, Zhang L, Hu Y, He M, Li Z, Cheng S, Yuan J, et al. Predicting gastric cancer response to anti-HER2 therapy or anti-HER2 combined immunotherapy based on multi-modal data. Signal Transduct Target Ther. 2024;9(1):222. 39183247 10.1038/s41392-024-01932-y PMC11345439

[37] Li YJ, Chou HH, Lin PC, Shen MR, Hsieh SY. A novel deep learning-based algorithm combining histopathological features with tissue areas to predict colorectal cancer survival from whole-slide images. J Transl Med. 2023;21(1):731. 37848862 10.1186/s12967-023-04530-8 PMC10580604

[38] Can Cui HL, Liu Q, Deng R, Asad Z, Wang Y, Zhao S, Yang H, Landman BA, Huo Y. Survival prediction of brain cancer with incomplete radiology, pathology, genomic, and demographic data. Cham: Springer; 2022.

[39] Wu X, Li Y, Chen J, Chen J, Zhang W, Lu X, Zhong X, Zhu M, Yi Y, Bu H. Multimodal recurrence risk prediction model for HR+/HER2- early breast cancer following adjuvant chemo-endocrine therapy: Integrating pathology image and clinicalpathological features. Breast Cancer Res. 2025;27(1):27. 40148997 10.1186/s13058-025-01968-0 PMC11951786

[40] Lu MY, Chen B, Williamson DFK, Chen RJ, Liang I, Ding T, Jaume G, Odintsov I, le LP, Gerber G, et al. A visual-language foundation model for computational pathology. Nat Med. 2024;30(3):863–874. 38504017 10.1038/s41591-024-02856-4 PMC11384335

[41] Chen RJ, Ding T, Lu MY, Williamson DFK, Jaume G, Song AH, Chen B, Zhang A, Shao D, Shaban M, et al. Towards a general-purpose foundation model for computational pathology. Nat Med. 2024;30(3):850–862. 38504018 10.1038/s41591-024-02857-3 PMC11403354

[42] Graham S, Vu QD, Raza SEA, Azam A, Tsang YW, Kwak JT, Rajpoot N. Hover-Net: Simultaneous segmentation and classification of nuclei in multi-tissue histology images. Med Image Anal. 2019;58: Article 101563. 31561183 10.1016/j.media.2019.101563

[43] Ankit Pal MS. OpenBioLLMs: Advancing open-source large language models for healthcare and life sciences. Hugging Face repository. 2024.

[44] Luo R, Sun L, Xia Y, Qin T, Zhang S, Poon H, Liu TY. BioGPT: Generative pre-trained transformer for biomedical text generation and mining. Brief Bioinform. 2022;23(6): Article bbac409. 36156661 10.1093/bib/bbac409

[45] Chakraborty S, Bisong E, Bhatt S, Wagner T, Elliott R, Mosconi F. BioMedBERT: A pre-trained biomedical language model for QA and IR. In: *Proceedings of the 28th International Conference on Computational Linguistics*. Barcelona (Spain): International Conference on Computational Linguistics; 2020. p. 669–679.

[46] Jacob Devlin M-WC, Lee K, Toutanova K. BERT: Pre-training of deep bidirectional transformers for language understanding. In: *Proceedings of the 2019 Conference of the North American Chapter of the Association for Computational Linguistics: Human Language Technologies, Volume 1 (Long and Short Papers)*. Minneapolis (MN): Association for Computational Linguistics; 2019. p. 4171–4186.

[47] Song AH, Chen RJ, Jaume G, Vaidya AJ, Baras AS, Mahmood F. Multimodal prototyping for cancer survival prediction. In: *Proceedings of the 41st International Conference on Machine Learning (ICML'24), Vol. 235*. JMLR.org. p. 46050–46073. *Vienna, Austria*. 2024.

