# Supplementary material for: Multimodal Deep Learning with Routine Clinical Data for Recurrence Risk Stratification in HR+/HER2− Early Breast Cancer
Source: Research (Wash D C). 2026 Mar 30;9:1136. doi: 10.34133/research.1136 (PMC13033830; doi:10.34133/research.1136)
Supplement: Supplementary 1 — Tables S1 to S3 Figs. S1 to S11 [file research.1136.f1.pdf]

Supplementary Information for

Multimodal deep learning with routine clinical data for recurrence risk stratification in HR+/HER2- early breast cancer

Xiaoyan Wu et al.

* Corresponding author: Yuhao Yi, yuhaoyi@scu.edu.cn

This PDF file includes:

Figs. S1 to S11
Tables S1 to S3

Table S1. Clinical characteristic of patients in the WCH cohort

Characteristic	N = 768[1]	No recurrence N = 643[1]	Recurrence N = 125[1]	P[2] value
Age (years)				0.34
Median (Range)	47 (25, 78)	47 (25, 78)	47 (30, 77)	
Tumor size				0.013
≤ 2cm	339 (44%)	297 (46%)	42 (34%)	
> 2cm	429 (56%)	346 (54%)	83 (66%)	
LN status				<0.001
-	323 (42%)	284 (44%)	39 (31%)	
1-3	286 (37%)	248 (39%)	38 (30%)	
≥ 4	159 (21%)	111 (17%)	48 (38%)	
Clinical stage				<0.001
Ⅰ	166 (22%)	147 (23%)	19 (15%)	
Ⅱ	438 (57%)	381 (59%)	57 (46%)	
Ⅲ	164 (21%)	115 (18%)	49 (39%)	
HER2 status				0.92
0	203 (26%)	169 (26%)	34 (27%)	
Low	565 (74%)	474 (74%)	91 (73%)	
Molecular subtype				0.73
Luminal A	160 (21%)	132 (21%)	28 (22%)	
Luminal B	608 (79%)	511 (79%)	97 (78%)	
Grade				0.004
1	33 (4.3%)	31 (4.8%)	2 (1.6%)	
2	444 (58%)	384 (60%)	60 (48%)	
3	291 (38%)	228 (35%)	63 (50%)	
ER				0.25
Median (Range)	0.90 (0.10, 1.00)	0.90 (0.10, 1.00)	0.90 (0.10, 1.00)	
PR				<0.001
Median (Range)	0.70 (0.00, 1.00)	0.75 (0.00, 1.00)	0.70 (0.00, 1.00)	
Ki67				0.96
Median (Range)	0.22 (0.01, 0.95)	0.20 (0.02, 0.95)	0.25 (0.01, 0.80)	
RFS (month)				<0.001
Median (Range)	84 (6, 174)	92 (41, 174)	47 (6, 136)	
OS (month)				<0.001
Median (Range)	88 (17, 174)	92 (41, 174)	78 (17, 168)	

1 n (%); 2 Kruskal-Wallis rank sum test; Pearson's Chi-squared test

Table S2. Model Performance with Different Query Vector Combinations in the Intra-Modality Cross-Attention Mechanism

Modal	WSI Features (Q)			US Features (Q)			Clinical	C-index (Δ)
	Deep	Morph	Top	Deep	Omic	Text		
WSI-Clinical	✓						✓	**0.800 (Ref)**
		✓					✓	0.560 (-0.24)
			✓				✓	0.615 (-0.185)
US-Clinical				✓			✓	**0.586 (Ref)**
					✓		✓	0.52 (-0.066)
						✓	✓	0.488 (-0.098)

Deep, deep learning-based image features extracted from WSIs/US; Morph, hand-crafted morphological features; Top, graph-based spatial topological features derived from cell-cell relationships; Omic, quantitative radiomics features extracted from ultrasound tumor regions using the PyRadiomics library; Text, features derived from text ultrasound diagnostic reports.

Table S3. Detailed description of single-cell morphological and topological features

Feature name	Category	Description
Area	Morphological	Total pixels in the target area, reflecting the actual size of the nucleus.
AreaBbox	Morphological	The area of the minimum bounding rectangle (bounding box) fully enclosing the target.
CellEccentricities	Morphological	The deviation of the cell shape from a perfect ellipse.
Circularity	Morphological	Degree of closeness to a perfect circle, with smaller values indicating greater irregularity.
Elongation	Morphological	Aspect ratio (long axis to short axis), indicating structural elongation or narrowness.
Extent	Morphological	Area-to-bounding box ratio, indicating the efficiency of target occupancy within the bounding box.
MajorAxisLength	Morphological	Length of the major axis of the fitted ellipse, representing the maximum diameter of the target.
MinorAxisLength	Morphological	Length of the Minor axis of the fitted ellipse, representing the minimum diameter of the target.
Perimeter	Morphological	Total length of the target boundary, used for analyzing shape complexity.
Solidity	Morphological	Area/Convex Area ratio, indicating the degree of surface concavity.
CurvMean	Morphological	Mean boundary curvature, describing the overall bending degree of the contour.
CurvMax	Morphological	Maximum boundary curvature, indicating the sharpest convex protrusion.
CurvMin	Morphological	Minimum boundary curvature, identifying the sharpest concave region.
CurvStd	Morphological	Boundary curvature fluctuation intensity, with higher values indicating more abrupt local shape variations.
ASM	Morphological	Sum of squared elements in GLCM, indicating the uniformity of image gray-level distribution.
Contrast	Morphological	Measuring the degree of intensity variation between adjacent pixels.

Correlation	Morphological	Quantifying the linear dependency between pixel pairs.
Entropy	Morphological	Reflecting the randomness or complexity of image texture.
Homogeneity	Morphological	Quantifying the concentration of elements near the GLCM diagonal, indicating local gray-level similarity.
IntensityMean	Morphological	Mean nuclear pixel intensity, representing the average grayscale value of all pixels within the nucleus.
IntensityStd	Morphological	Standard deviation of nuclear pixel intensity, reflecting the dispersion degree of grayscale values within the nucleus.
IntensityMax	Morphological	Maximum nuclear pixel intensity, indicating the highest grayscale value within the nucleus.
IntensityMin	Morphological	Minimum nuclear pixel intensity, indicating the lowest grayscale value within the nucleus.
Nsubgraph	Topological	Number of nuclei in the subgraph.
Degrees	Topological	Number of edges connected to the nucleus.
Coreness	Topological	The coreness of a nucleus is k if it is a member of the k-core but not a member of the (k+1)core.
Eccentricity	Topological	The maximum of the shortest distance from this nucleus to all other nuclei in the graph.
Eccentricity_normed	Topological	The normalized value of the eccentricity.
Harmonic Centrality	Topological	Measures the accessibility of a nucleus from other nuclei, calculated as the average inverse distance to all other nuclei.
Closeness	Topological	Quantifies the accessibility of a given nucleus to all others
Betweenness	Topological	Nuclear betweenness centrality measures the bridging role of a nucleus within the graph.
Betweenness_normed	Topological	The normalized value of the betweenness.
Clustering Coefficien	Topological	Measuring the probability that two neighboring nuclei of a given nucleus are also interconnected.
MinEdgeLength	Topological	The minimum length of the edges of the nucleus. If there is no edge connected with the nucleus, the value is set as 100 pixels, which is the upper limit of distance between two connected nuclei.

MeanEdgeLength	Topological	The mean length of the edges of the nucleus. If there is no edge connected with the nucleus, the value is set as 100 pixels, which is the upper limit of distance between two connected nuclei.

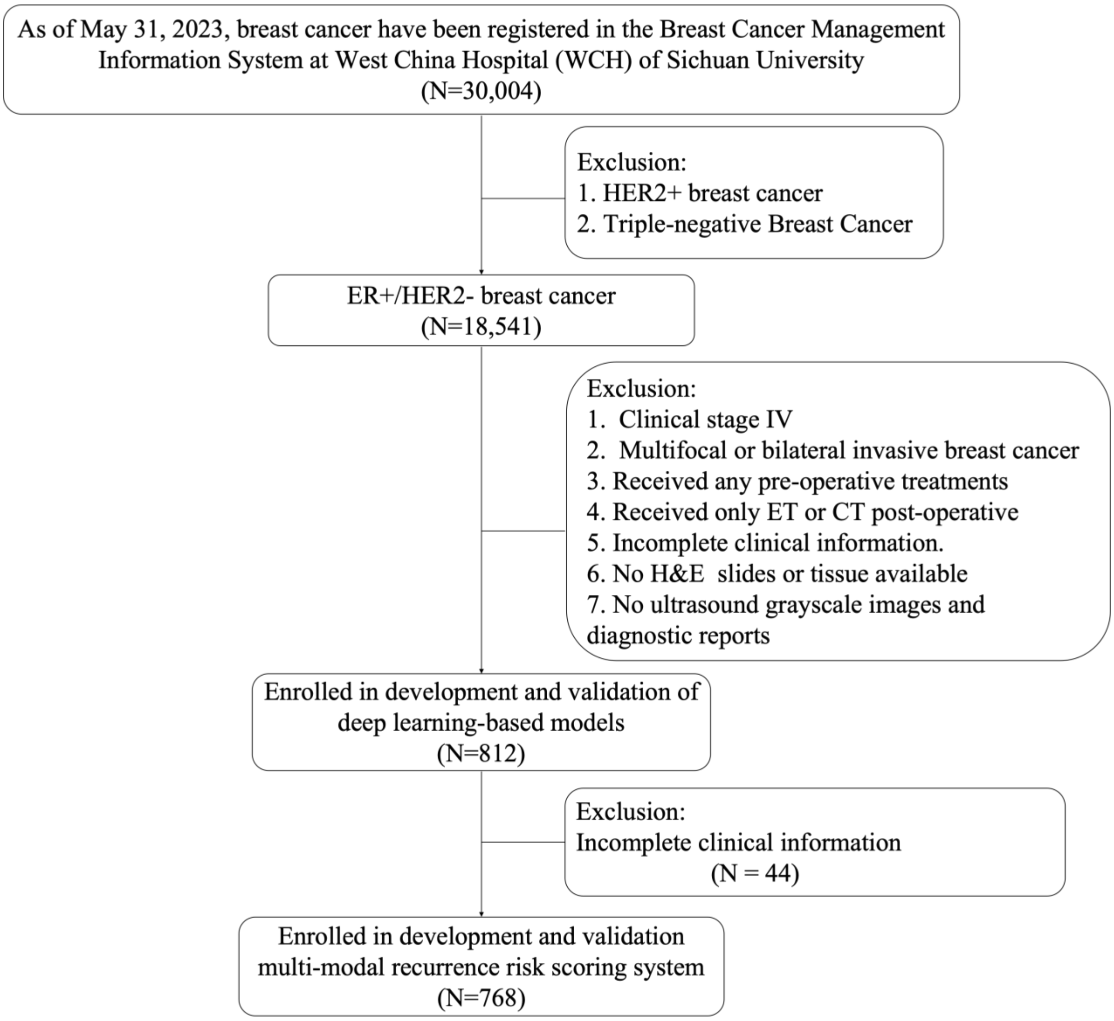

Figure S1. Patient Recruitment Workflow for the WCH Cohort

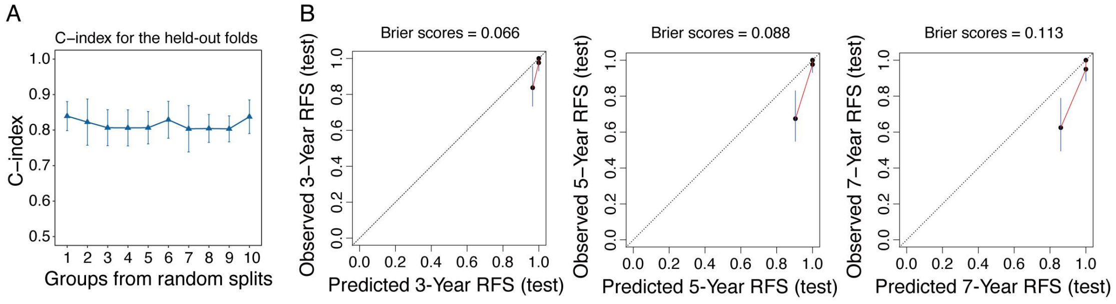

Figure S2. Performance of the MRRP model. A C-index and 95% confidence interval (CI) for the held-out folds from each of the 10 repeated five-fold cross-validations using different random seeds. B In the test set, time-dependent calibration curves and Brier scores at 3, 5, and 7 years.

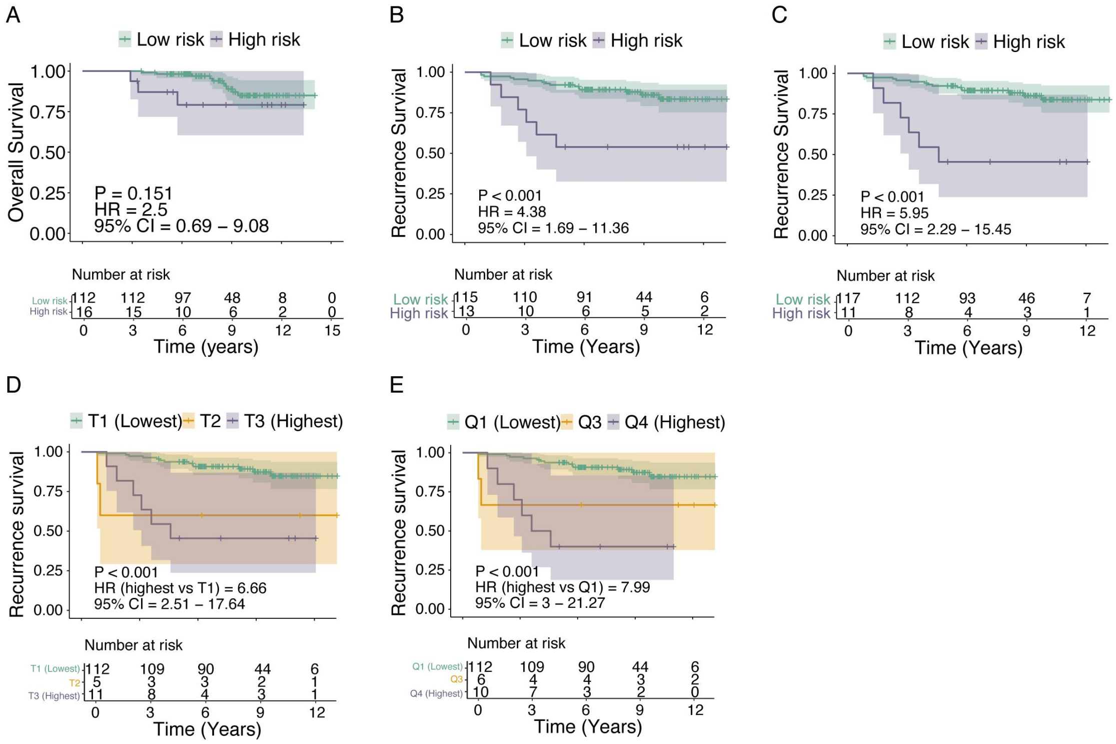

Figure S3. Performance of the MRRP model. Kaplan-Meier curves for (A) overall survival (OS) and (B-E) recurrence-free survival (RFS) based on MRRP stratified risk groups. Patients were stratified into high-risk (purple) and low-risk (green) using different cut-offs of the MRRP score: median (A), 60th percentile (B), 70th percentile (C), tertiles (D), and quartiles (E). Differences between groups were assessed by the two-sided log-rank test (P < 0.05).

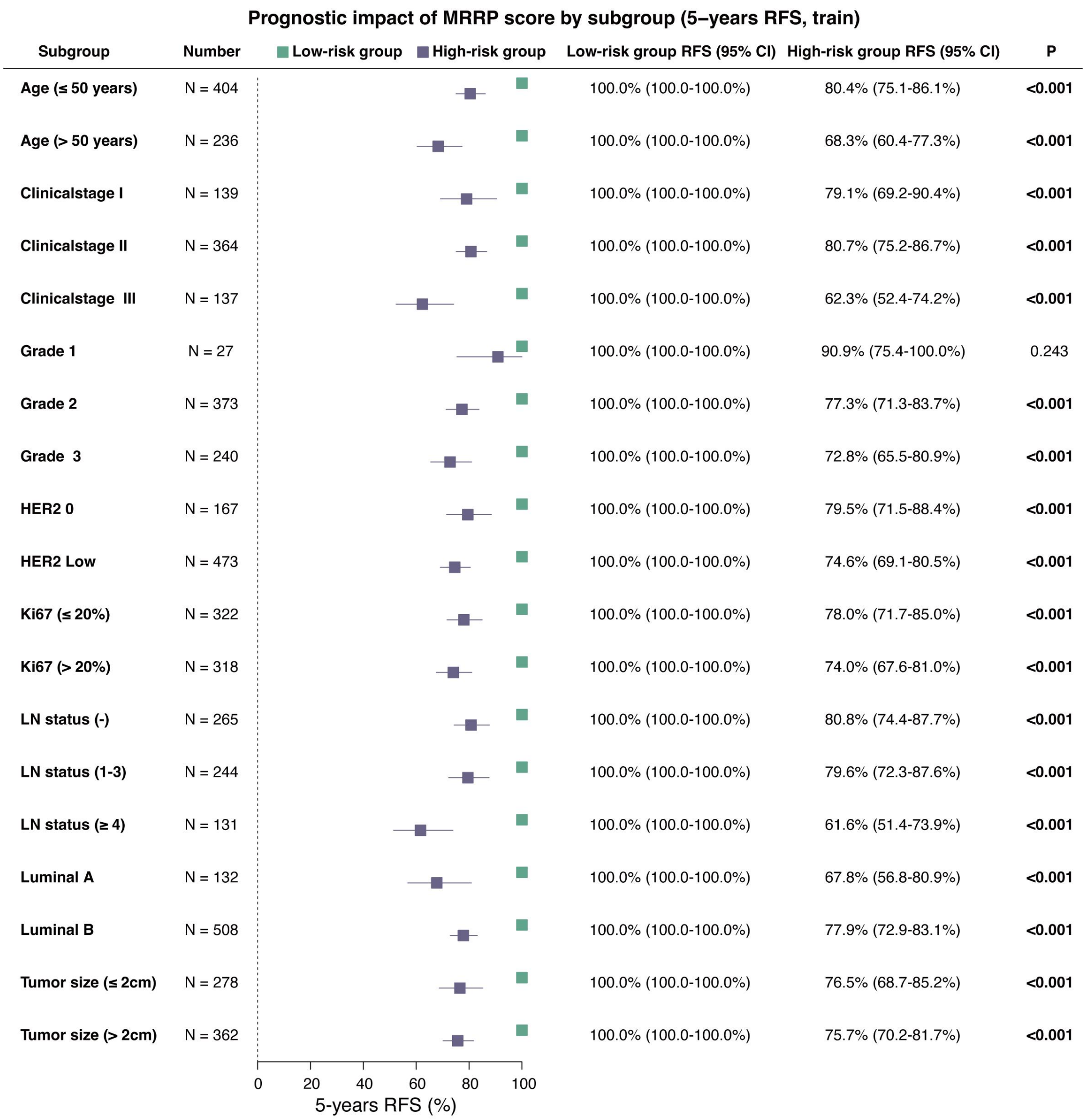

Figure S4. Subgroup performance of the MRRP model in the training set. Prognostic impact of MRRP score-based risk stratification across predefined clinicopathologic subgroups. For each subgroup, patients were classified into low-risk and high-risk groups according to the MRRP score cut-off, and the estimated 5-year recurrence-free survival (RFS) with 95% confidence intervals (CIs) is shown. N denotes the number of patients in each subgroup. P values were calculated using the two-sided log-rank test.

Prognostic impact of MRRP score by subgroup (5–years RFS, test)

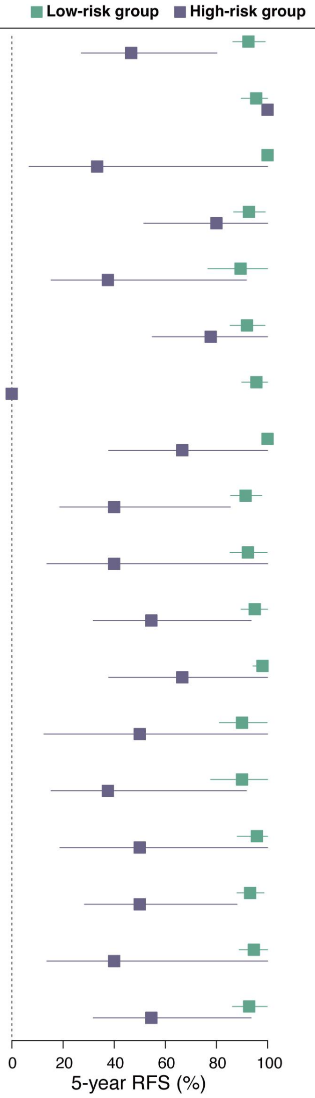

Subgroup	Number	Low-risk group RFS (95% CI)	High-risk group RFS (95% CI)	P
**Age (≤ 50 years)**	N = 82	92.5% (86.5-99.0%)	46.7% (27.2-80.2%)	**<0.001**
**Age (> 50 years)**	N = 46	95.6% (89.7-100.0%)	100.0% (100.0-100.0%)	0.637
**Clinicalstage I**	N = 27	100.0% (100.0-100.0%)	33.3% (6.7-100.0%)	**<0.001**
**Clinicalstage II**	N = 74	92.7% (86.8-99.1%)	80.0% (51.6-100.0%)	0.665
**Clinicalstage III**	N = 27	89.5% (76.7-100.0%)	37.5% (15.3-91.7%)	**0.029**
**Grade 2**	N = 71	91.9% (85.4-99.0%)	77.8% (54.9-100.0%)	0.320
**Grade 3**	N = 51	95.7% (89.9-100.0%)	0.0% (0.0-0.0%)	**<0.001**
**HER2 0**	N = 36	100.0% (100.0-100.0%)	66.7% (37.9-100.0%)	0.090
**HER2 Low**	N = 92	91.4% (85.6-97.7%)	40.0% (18.7-85.5%)	**<0.001**
**Ki67 (≤ 20%)**	N = 57	92.3% (85.3-99.8%)	40.0% (13.7-100.0%)	**<0.001**
**Ki67 (> 20%)**	N = 71	95.0% (89.6-100.0%)	54.5% (31.8-93.6%)	**0.002**
**LN status (-)**	N = 58	98.0% (94.3...100.0%)	66.7% (37.9-100.0%)	**0.003**
**LN status (1-3)**	N = 42	90.0% (81.2-99.8%)	50.0% (12.5-100.0%)	0.124
**LN status (≥ 4**	N = 28	90.0% (77.8-100.0%)	37.5% (15.3-91.7%)	**0.021**
**Luminal A**	N = 28	95.8% (88.2-100.0%)	50.0% (18.8-100.0%)	**0.002**
**Luminal B**	N = 100	93.2% (88.0-98.6%)	50.0% (28.4-88.0%)	**<0.001**
**Tumor size (≤ 2cm)**	N = 61	94.6% (88.9-100.0%)	40.0% (13.7-100.0%)	**<0.001**
**Tumor size (> 2cm)**	N = 67	92.8% (86.3-99.9%)	54.5% (31.8-93.6%)	**0.008**

Figure S5 Subgroup performance of the MRRP model in the test set. Prognostic impact of MRRP score-based risk stratification across predefined clinicopathologic subgroups. For each subgroup, patients were classified into low-risk and high-risk groups according to the MRRP score cut-off, and the estimated 5-year recurrence-free survival (RFS) with 95% confidence intervals (CIs) is shown. N denotes the number of patients in each subgroup. P values were calculated using the two-sided log-rank test.

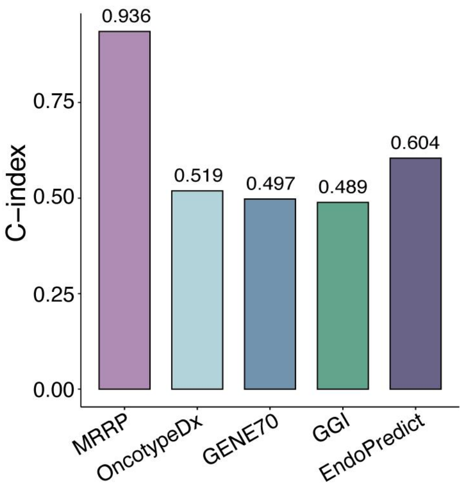

Figure S6. Comparison of prognostic performance between MRRP and multigene signatures in the transcriptomic subset. Bar plot of C-index values for recurrence prediction in a subset of 68 patients with available transcriptomic data. Multigene signatures were computed using the genefu package according to the procedures described in our previous work, including Oncotype DX, MammaPrint (GENE70), GGI, and EndoPredict.

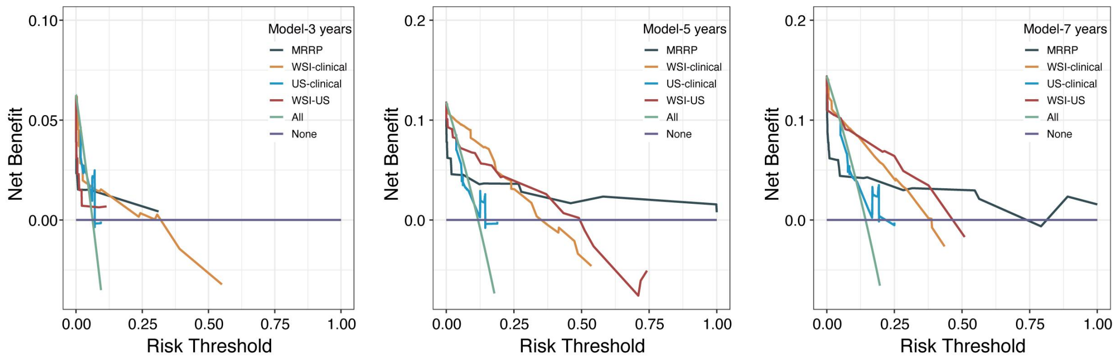

Figure S7. Decision-curve analysis of the MRRP model. Decision-curve analysis (DCA) curves at 3, 5, and 7 years evaluating the net benefit of the MRRP score across a range of threshold probabilities, compared with the WSI-clinical, US-clinical, and WSI-US models, as well as the treat-all and treat-none strategies.

A. High risk patient

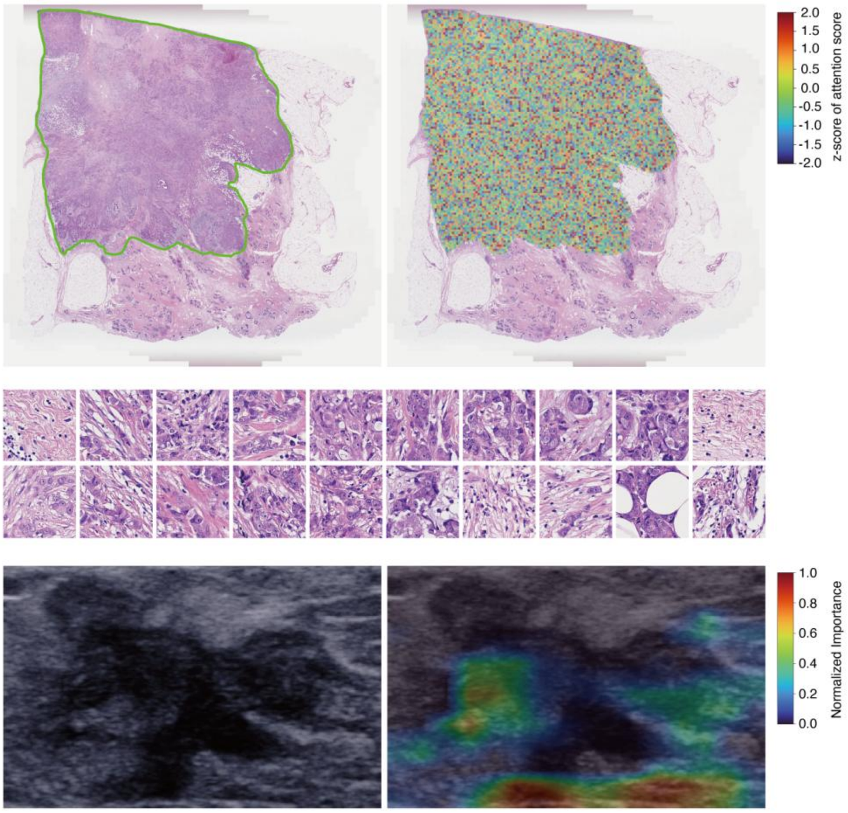

B. Low risk patient

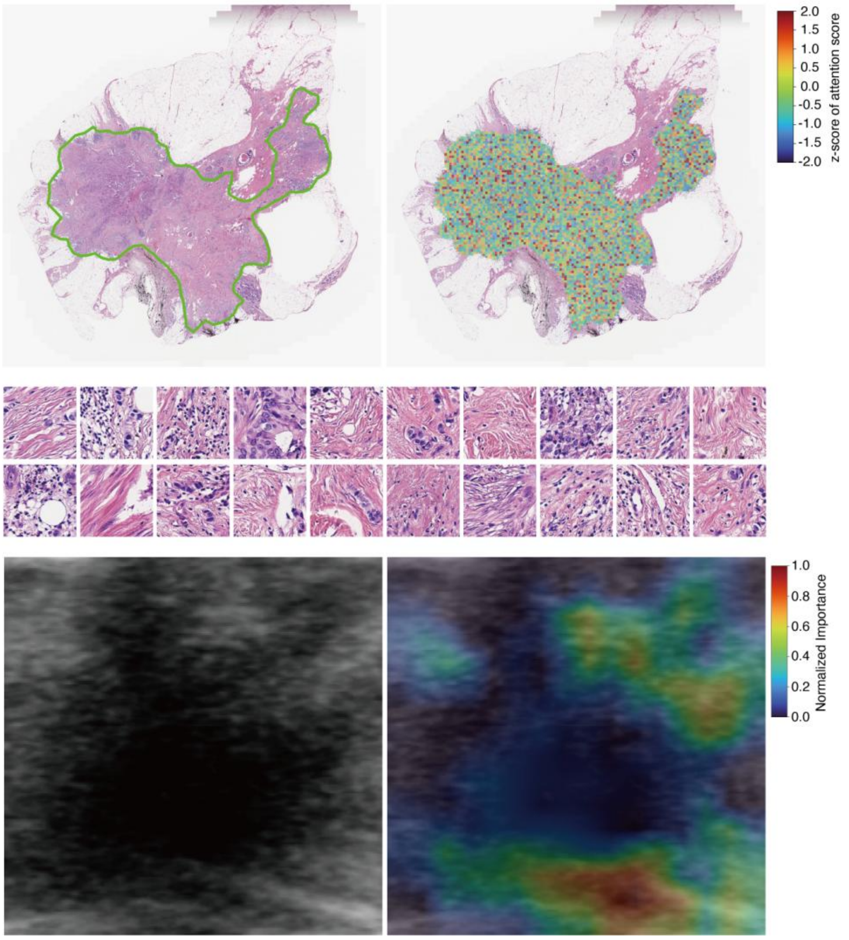

Figure S8. Representative interpretability visualizations for the MRRP model. A-B. Representative high- and low-risk patients showing whole-slide image (WSI) and ultrasound heatmaps, along with the top 20 most-attended WSI patches.

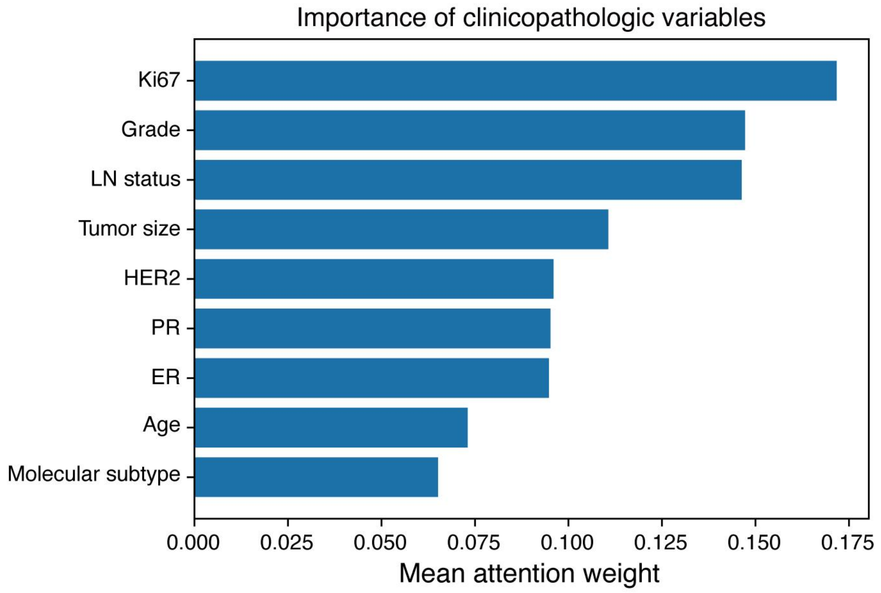

Figure S9. Importance of clinicopathologic variables in the MRRP model. Bar plot showing the mean attention weight assigned to each clinicopathologic variable, used to rank variable importance.

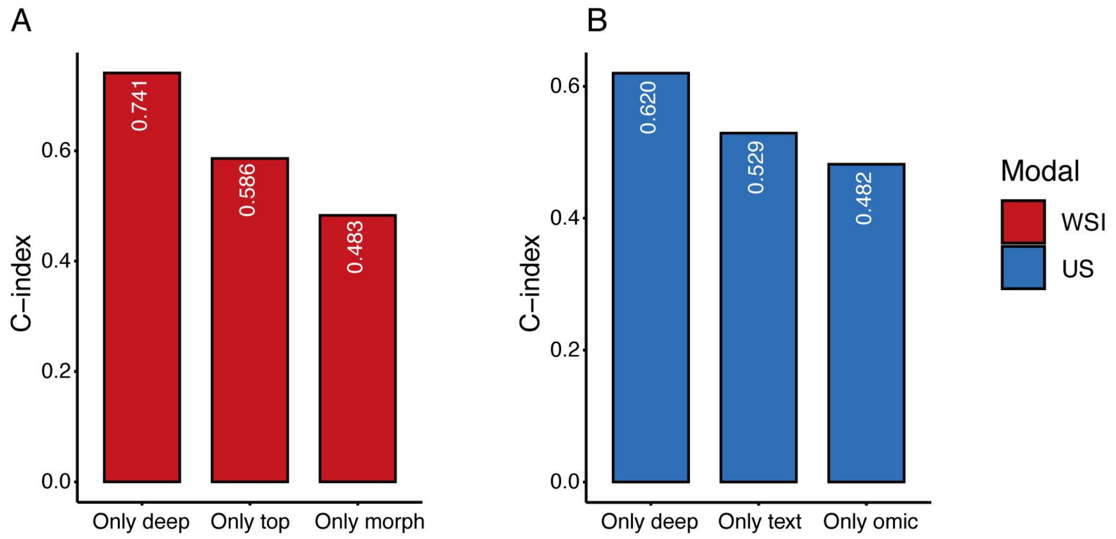

Figure S10. Impact of Different Intra-modality Features on Model Performance. A. Bar chart showing the C-index of single-feature models within the WSI single-modality on the test set. B. Bar chart showing the C-index of single-feature models within the US single-modality on the test set.

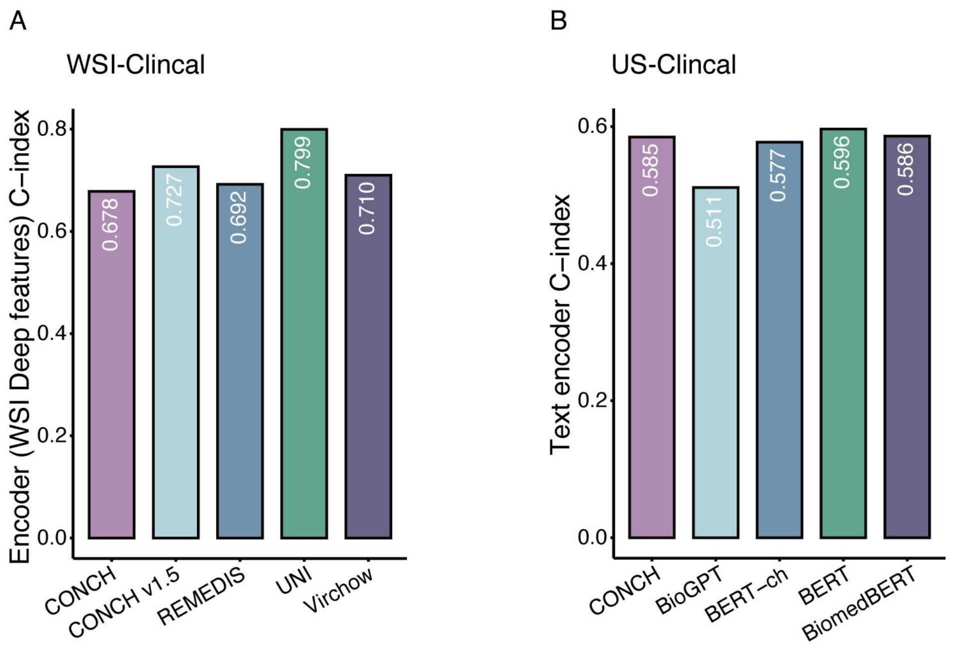

Figure S11. Impact of Pretrained Encoders on Model Performance. A. Bar chart showing the C-index of different WSI pretrained encoders in the pathology-clinical model. B. Bar chart showing the C-index of different large pretrained models in the ultrasound-clinical model.
